# Comparative analysis of stress-induced calcium signals in the crop species barley and the model plant *Arabidopsis thaliana*

**DOI:** 10.1186/s12870-022-03820-5

**Published:** 2022-09-17

**Authors:** Maya Giridhar, Bastian Meier, Jafargholi Imani, Karl-Heinz Kogel, Edgar Peiter, Ute C. Vothknecht, Fatima Chigri

**Affiliations:** 1 Plant Cell Biology, IZMB, University of Bonn, Kirschallee 1, D-53115 Bonn, Germany; 2 Institute of Agricultural and Nutritional Sciences, Faculty of Natural Sciences III, Martin Luther University Halle-Wittenberg, Betty Heimann Str. 3, D-06120 Halle (Saale), Germany; 3 Research Centre for BioSystems, Land Use and Nutrition (IFZ), Institute for Phytopathology, Justus Liebig University Gießen, Heinrich-Buff-Ring 26-32, D-35392 Gießen, Germany

**Keywords:** Aequorin, *Arabidopsis thaliana*, barley, Ca^2+^ signalling, Ca^2+^ signature, *Hordeum vulgare*, Drought stress, flagellin 22, Salt stress, Oxidative stress

## Abstract

**Background:**

Plants are continuously exposed to changing environmental conditions and biotic attacks that affect plant growth. In crops, the inability to respond appropriately to stress has strong detrimental effects on agricultural production and yield. Ca^2+^ signalling plays a fundamental role in the response of plants to most abiotic and biotic stresses. However, research on stimulus-specific Ca^2+^ signals has mostly been pursued in *Arabidopsis thaliana*, while in other species these events are little investigated .

**Results:**

In this study, we introduced the Ca^2+^ reporter-encoding gene *APOAEQUORIN* into the crop species barley (*Hordeum vulgare*). Measurements of the dynamic changes in [Ca^2+^]_cyt_ in response to various stimuli such as NaCl, mannitol, H_2_O_2_, and flagellin 22 (flg22) revealed the occurrence of dose- as well as tissue-dependent [Ca^2+^]_cyt_ transients. Moreover, the Ca^2+^ signatures were unique for each stimulus, suggesting the involvement of different Ca^2+^ signalling components in the corresponding stress response. Alongside, the barley Ca^2+^ signatures were compared to those produced by the phylogenetically distant model plant Arabidopsis. Notable differences in temporal kinetics and dose responses were observed, implying species-specific differences in stress response mechanisms. The plasma membrane Ca^2+^ channel blocker La^3+^ strongly inhibited the [Ca^2+^]_cyt_ response to all tested stimuli, indicating a critical role of extracellular Ca^2+^ in the induction of stress-associated Ca^2+^ signatures in barley. Moreover, by analysing spatio-temporal dynamics of the [Ca^2+^]_cyt_ transients along the developmental gradient of the barley leaf blade we demonstrate that different parts of the barley leaf show quantitative differences in [Ca^2+^]_cyt_ transients in response to NaCl and H_2_O_2_. There were only marginal differences in the response to flg22, indicative of developmental stage-dependent Ca^2+^ responses specifically to NaCl and H_2_O_2_.

**Conclusion:**

This study reveals tissue-specific Ca^2+^ signals with stimulus-specific kinetics in the crop species barley, as well as quantitative differences along the barley leaf blade. A number of notable differences to the model plants Arabidopsis may be linked to different stimulus sensitivity. These transgenic barley reporter lines thus present a valuable tool to further analyse mechanisms of Ca^2+^ signalling in this crop and to gain insights into the variation of Ca^2+^-dependent stress responses between stress-susceptible and -resistant species.

**Supplementary Information:**

The online version contains supplementary material available at 10.1186/s12870-022-03820-5.

## Background

Plants as sessile organisms are exposed to constantly changing conditions in their environment that require short-term acclimation responses enabling them to fulfil their lifecycle. Thus, it is crucial to understand mechanisms of stress response to maintain and improve crop production, especially in the face of increasing global climate changes [1]. Interestingly, plants show different sensitivity to environmental factors depending on their genetic make-up, which defines adaptive mechanisms enabling different degrees of stress tolerance [2]. In the last decades, the model plant *Arabidopsis thaliana* has been extensively studied to decipher the molecular basis of stress tolerance; however, the study of more stress-tolerant species is more likely to yield relevant information.

Barley (*Hordeum vulgare* L.) is well-adapted to a wide range of environmental conditions and has a relatively high tolerance to drought and salinity compared to other crops and Arabidopsis [3–5]. With an acreage of ~ 50 million hectares, barley is the fourth-most cultivated cereal crop in the world after maize (*Zea mays*), rice (*Oryza sativa*), and wheat (*Triticum aestivum*), which makes it a versatile commodity in many different countries. Therefore, determining and understanding the biological mechanisms involved in barley stress responses are important for the improvement of this valuable crop and can also contribute to a better understanding of those mechanisms in other cereal crops.

Generally, plants respond to environmental stimuli by initiating signalling cascades that coordinate plant adaptive responses that contribute to stress tolerance [6, 7]. These cascades comprise a collection of signalling compounds and downstream signalling events, including posttranslational protein modifications and regulation of gene expression. Calcium (Ca^2+^) is a major signalling ion involved in responses to a wide range of abiotic and biotic stresses. As a basis for Ca^2+^ signalling, and due to its cytotoxicity, resting levels of the free Ca^2+^ concentration in the cytosol ([Ca^2+^]_cyt_) are maintained in the nanomolar range [8, 9]. The perception of stimuli and stresses induces transient increases in [Ca^2+^]_cyt_ with stimulus-specific spatio-temporal parameters such as frequency, amplitude and duration, defined as “Ca^2+^ signatures” [10, 11]. Several studies showed that this increase in [Ca^2+^]_cyt_ is one of the earliest signalling events in plants challenged by biotic elicitors [12–14], herbivory [15], as well as abiotic stimuli including salt and osmotic stress [16, 17], oxidative stress [18], light stress [19], or cold stress [12, 20].

The stimulus-induced increases of [Ca^2+^]_cyt_ result from the influx of Ca^2+^ via Ca^2+^-permeable channels, either from intracellular Ca^2+^ stores or across the plasma membrane from the apoplast. These elevations in [Ca^2+^]_cyt_ are in turn counteracted by the activity of Ca^2+^ transporters or ATPases extruding Ca^2+^ out of the cytosol to restore the low basal level of [Ca^2+^]_cyt_. In Arabidopsis, multiple families of Ca^2+^-permeable channels, transporters and pumps have been identified, each with several members [21]. However, the specific function and regulation of the individual proteins, as well as their combined action to shape Ca^2+^ signatures are still widely unknown [22].

The information encoded by Ca^2+^ signatures is generally detected and decoded by a toolkit of Ca^2+^ sensors, which in turn interact with and activate downstream targets to contribute to the stimulus-specific cellular response [23, 24]. In plants, these Ca^2+^ sensors are primarily calmodulins (CaMs), calmodulin-like proteins (CMLs), Ca^2+^-dependent protein kinases (CDPKs), as well as calcineurin B-like proteins (CBLs) and their interacting protein kinases (CIPKs) [25]. These sensors are encoded by large gene families with a plethora of known targets, underscoring the tremendous importance of Ca^2+^ signalling in plants [26, 27]. The interplay of Ca^2+^ signatures and Ca^2+^ sensors is supposed to confer the specificity and flexibility of Ca^2+^ signalling required to respond to various stimuli in a tissue- and development-dependent manner.

Since the 1990s, the bioluminescent Ca^2+^ reporter aequorin, originating from the jellyfish *Aequoria victoria*, has enabled tremendous advances in the detection of Ca^2+^ signals in living plants [12] and is today still instrumental in our quest towards a mechanistic understanding of Ca^2+^ signal generation [28, 29]. The vast majority of such studies on Ca^2+^ signals in response to abiotic and biotic stimuli has been performed on Arabidopsis, and it is currently largely unknown how far plant species differ in this respect. Differences in Ca^2+^ responses to salt, oxidative stress, and pathogen-associated molecular patterns (PAMPs) have been found in studies comparing rice and Arabidopsis, possibly related to differences in their mechanisms to cope with the stresses [30, 31]. Signalling pathways in other cereals that hold monumental contributions to human and animal nutrition are widely uncharted.

In this study, the stable transformation of barley plants with *APOAEQUORIN* targeted to the cytosol enabled us to analyse [Ca^2+^]_cyt_ signals in this crop. The transgenic plants exposed to different abiotic stimuli (mannitol, NaCl, and H_2_O_2_) and the PAMP flg22 showed increases in [Ca^2+^]_cyt_ in a dose- and tissue-dependent manner. The spatio-temporal patterns of the cytosolic Ca^2+^ dynamics (Ca^2+^ signature) were unique for each stimulus. Furthermore, we revealed a conspicuous spatial heterogeneity of Ca^2+^ responses to some stimuli along the blade of barley leaves, indicating a dependence on the developmental stage of the cells. Compared to Arabidopsis notable differences in the barley Ca^2+^ signatures were observed. The comparison of Ca^2+^ signatures between barley and Arabidopsis, phylogenetically distant species with different stress tolerance, highlights the diversity in Ca^2+^ signals in higher plants and might help to reveal novel aspects in stress response mechanisms.

## Results

### Generation of transgenic barley lines expressing *APOAEQUORIN*

**Fig. 1:**
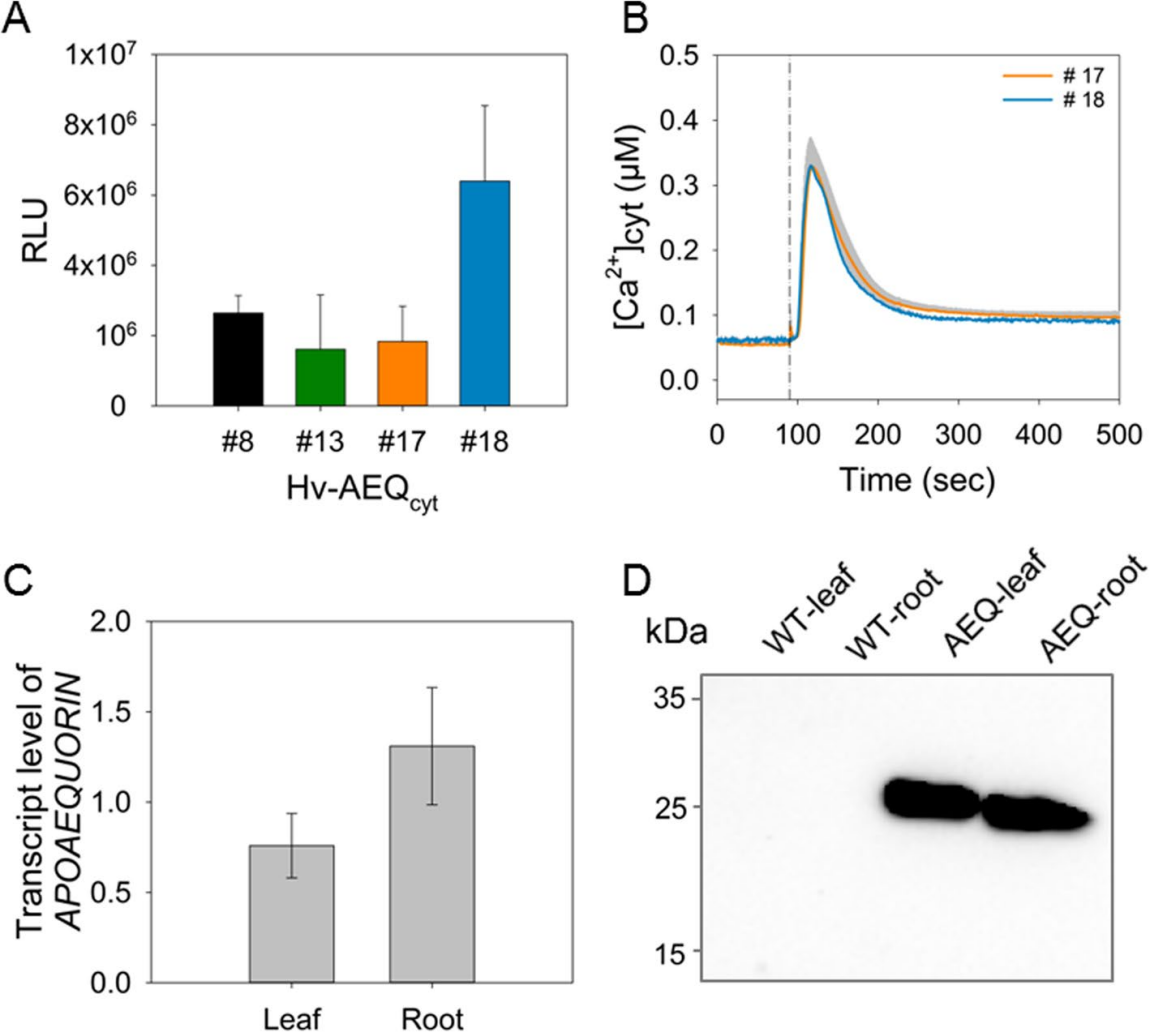
Selection and characterization of Hv-AEQ_cyt_ lines. **A** Ca^2+^-dependent photon release in relative light units (RLU) of different transgenic barley lines expressing the recombinant *APOAEQUORIN* (Hv-AEQ_cyt_ #8, #13, #17, and #18) after discharge of aequorin. **B** [Ca^2+^]_cyt_ of Hv-AEQ_cyt_ #17 and #18 in response to H_2_O_2_ (10 mM). The dashed line represents the time point of H_2_O_2_ injection. **C** Transcript level of *APOAEQUORIN* in leaves and roots of Hv-AEQ_cyt_ #18 determined against a cDNA dilution series and normalized to Actin (AY145451.1). **D** Abundance of aequorin in leaves and roots of Hv-AEQ_cyt_ #18 and wild type (WT) as negative control determined by immunodetection using a specific antibody against aequorin. Values represent means ± SE of three independent replicates

To monitor [Ca^2+^]_cyt_ changes in barley, transgenic lines expressing the genetically encoded Ca^2+^ sensor *APOAEQUORIN* under the control of the *ZmUBI1* promoter were developed and named Hv-AEQ_cyt_. Four independent transgenic lines (Hv-AEQ_cyt_ #8, #13, #17, and #18), based on the barley cultivar Golden Promise, were selected. To estimate the abundance of *APOAEQUORIN* in these lines, the aequorin-based luminescence was recorded in excised leaf tips (5 mm) after reconstitution with coelenterazine by injecting a discharge solution. Upon application of the discharge solution, all transgenic lines showed an increase in luminescence, with line #18 showing the highest intensity (Fig. 1A). The functionality of aequorin and the reliability of the conversion equation were further tested in the Hv-AEQ_cyt_ lines #18 and #17, which strongly differed in total luminescence. Upon injection of 10 mM H_2_O_2_, a rapid increase in [Ca^2+^]_cyt_ was observed in both lines with identical kinetic patterns (Fig. 1B), independent of the strength of the discharge in these lines. These results indicate that the transgenic barley lines carrying *APOAEQUORIN* can faithfully report changes in [Ca^2+^]_cyt_ and thus can be employed to investigate [Ca^2+^]_cyt_ signals in response to different stimuli. All further studies were then performed with line Hv-AEQ_cyt_ #18.

Quantitative RT-PCR analysis on RNA from leaves and roots of Hv-AEQ_cyt_ #18 confirmed that *APOAEQUORIN* is expressed in both tissues (Fig. 1C). In addition, western blot analysis using an aequorin-specific antibody detected the protein in leaves and roots of the transgenic plants (Fig. 1D; Fig. S1A). A photon-counting camera system was employed to record spatially resolved luminescence intensity of intact three-day-old seedlings. Upon application of discharge solution, luminescence was observed throughout shoot and roots (Fig. S1B-D), confirming the presences of APOAEQUORIN and its successful reconstitution to aequorin in both tissues.

**Fig. 2:**
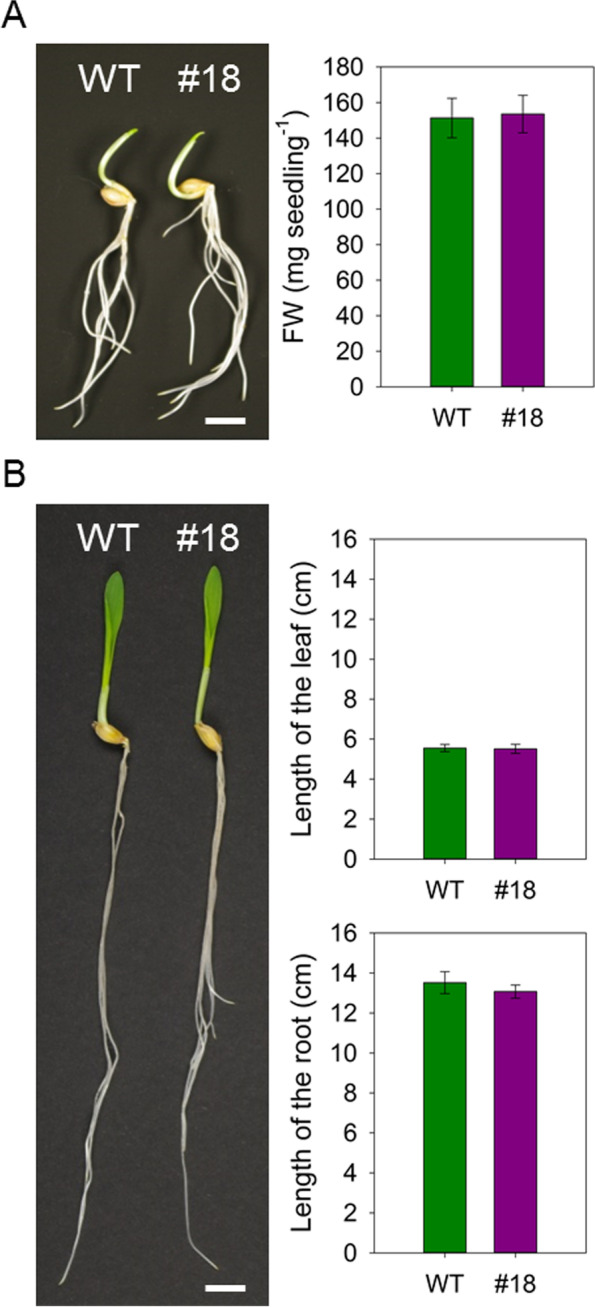
Seedlings of Hv-AEQcyt #18 shows no quantifiable differences to the wild type. **A** Visual phenotype of three-day-old barley wild type (WT) and transgenic (Hv-AEQ_cyt_ #18) plants grown under long-day conditions on vermiculite (left panel; bar = 1 cm) and fresh weight (FW) of those plants (right panel). **B** Visual phenotype of five-day old plants grown under the same conditions (left panel; bar = 1 cm) and length of leaf blades and roots of those plants (right panel). Values represent means ± SE of eight independent plants

The insertion of the *APOAEQUORIN* gene did not result in any notable phenotypic differences to the wild type in the transgenic plants, neither during early development nor maturity (Fig. 2, Fig. S2). The fresh weight of three-day-old seedlings, as well as the length of roots and leaf blades of five-day-old seedlings, was comparable between both genotypes (Fig. 2). Furthermore, there was no visible difference in spike development, seed set (Fig. S2A) or difference in thousand grain weight compared to wild type (Fig. S2B).

### [Ca^2+^]_cyt_ changes in barley and Arabidopsis induced by NaCl

To reveal stimuli-induced changes in [Ca^2+^]_cyt_, leaf and root tips of three-day-old Hv-AEQ_cyt_ #18 barley plants grown on vermiculite were analysed in a luminometer by the use of 96 well plates. For comparison, measurements were carried out on *APOAEQUORIN*-expressing Arabidopsis seedlings [20, 32]. Due to the developmental differences between barley and Arabidopsis, the entire shoot and root of five-day-old Arabidopsis seedlings, grown under the same conditions, were used for the measurements.

**Fig. 3:**
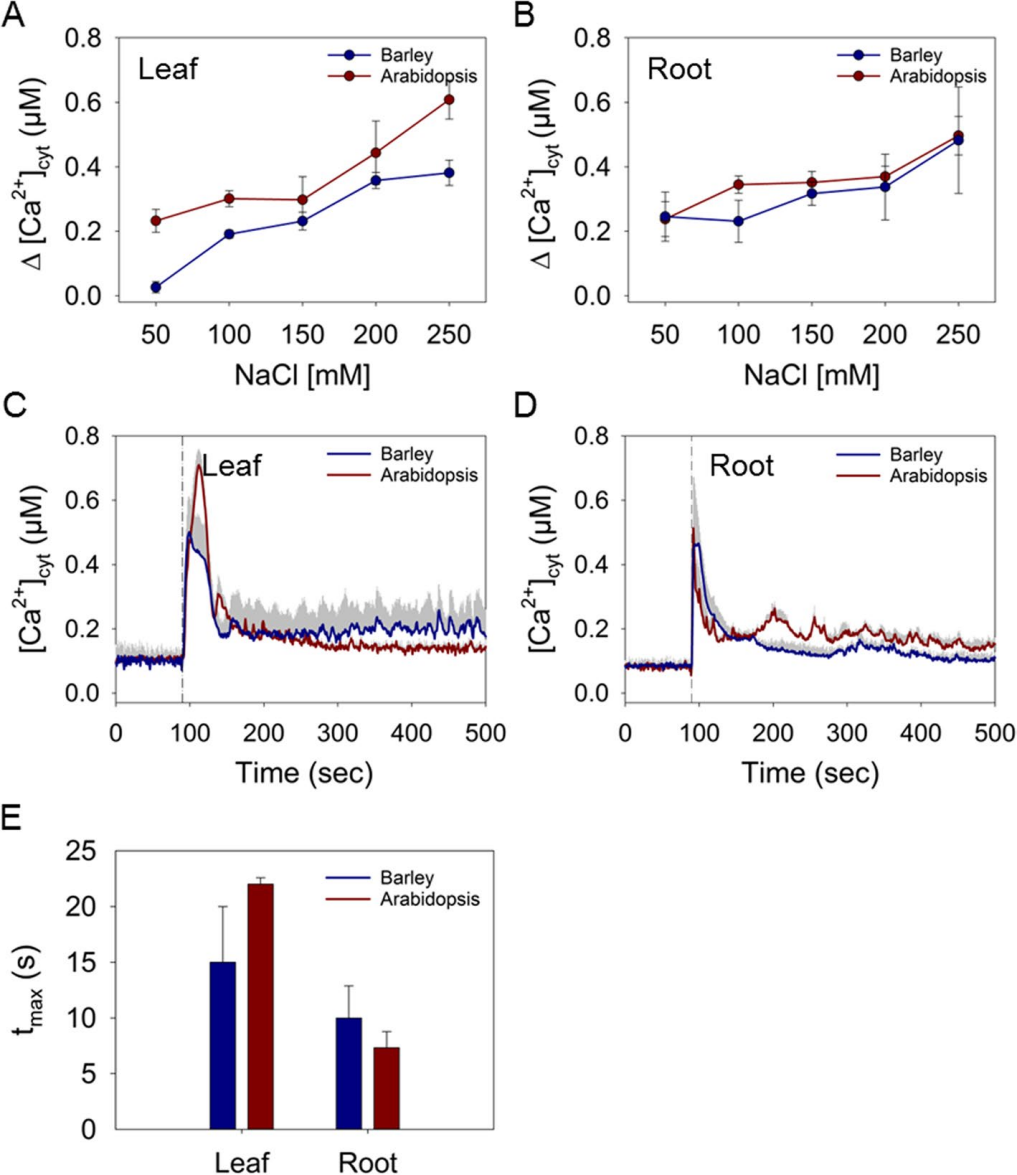
Induction of Ca^2+^ signals in response to NaCl. Maximal Δ[Ca^2+^]_cyt_ in barley and Arabidopsis leaf (**A**) and root (**B**) tissue induced by different concentrations of NaCl. Time courses of changes in [Ca^2+^]_cyt_ induced by 250 mM NaCl in leaf (**C**) and root (**D**) tissues. The dashed lines represent the time point of injection of the treatment. **E** Time to reach the maximal increase of [Ca^2+^]_cyt_ after injection of 250 mM NaCl in seconds (s). Values represent means ± SE of three independent replicates

NaCl-induced increases in [Ca^2+^]_cyt_ were observed in the leaf and root of barley in a dose-dependent manner (Fig. 3A, B). NaCl induced a fast and sharp increase of [Ca^2+^]_cyt_ in both tissues that quickly declined within 50 s to nearly resting levels (Fig. S3A, B). The amplitude of the [Ca^2+^]_cyt_ increase (Δ[Ca^2+^]_cyt_) varied depending on NaCl concentration and tissue. Already 50 mM NaCl induced obvious increases in [Ca^2+^]_cyt_ in both tissues, which was more pronounced in the root (Fig. 3A, B). The response became stronger with increasing NaCl concentration, reaching a maximum peak at about 0.5 μM Δ[Ca^2+^]_cyt_ upon treatment with 250 mM NaCl, which was more prolonged in leaf tips compared to the root (Fig. 3B, C). The NaCl treatments also induced dose-dependent [Ca^2+^]_cyt_ rises in Arabidopsis, but with dynamics notably different to those in barley (Fig. 3A - D; Fig. S3C, D). In Arabidopsis the application of 250 mM NaCl evoked a higher increase in [Ca^2+^]_cyt_ with a peak at about 0.7 μM Δ[Ca^2+^]_cyt_ in leaves (Fig. 3 A, C), while in roots the increase was lower and comparable to that in barley (Fig. 3B, D). The time to reach the maximal [Ca^2+^]_cyt_ after application of 250 mM NaCl differed significantly between the tissues but not between the two species (Fig. 3E). The maximal increase was reached after 15 to 22 s in Arabidopsis and barley leaves, while in root tissues this point was reached already after 7 to 10 s. Secondary small [Ca^2+^]_cyt_ elevations were recorded in roots of Arabidopsis that were not seen in barley roots (Fig. S3B, D). This heterogeneity in Ca^2+^ responses between shoot and root has been already described in Arabidopsis, and it was suggested to result from the different cell populations in the tissues [33, 34]. The differences in NaCl-induced [Ca^2+^]_cyt_ signals between the two species could be due to the fact that only root and leaf tips were used in barley. However, they also likely impinge on downstream processes of salt signalling and may be related to the differential responsiveness of Arabidopsis and barley to salt stress [35].

### [Ca^2+^]_cyt_ changes in barley and Arabidopsis induced by mannitol

**Fig. 4:**
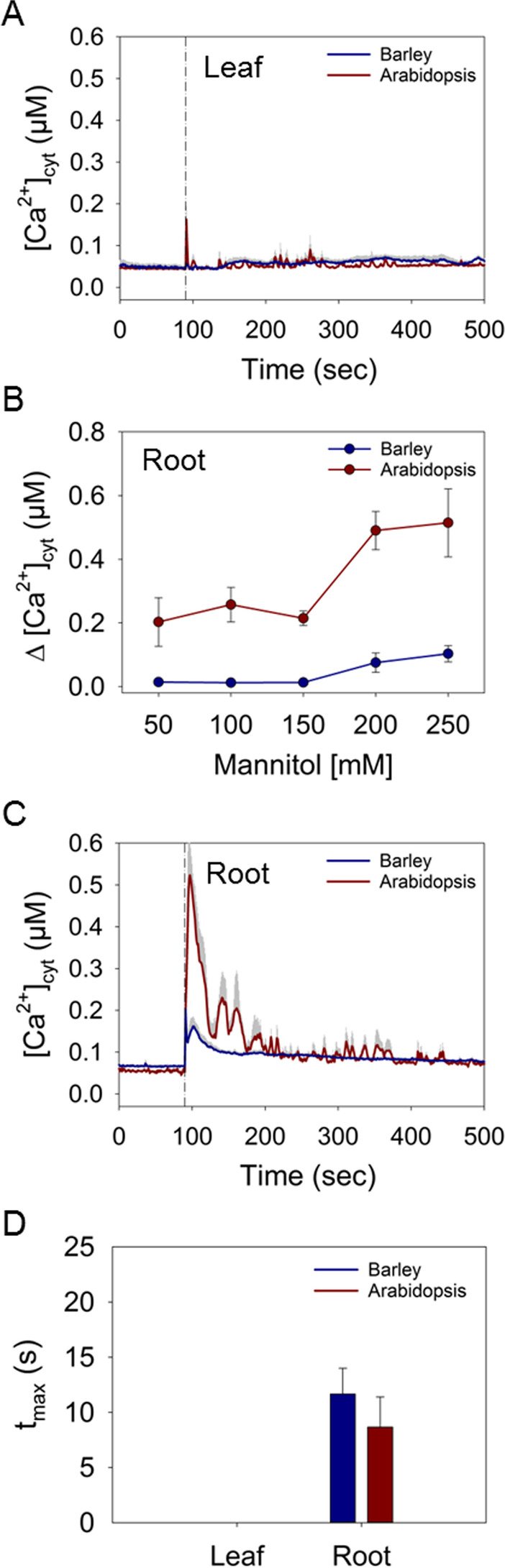
Induction of Ca^2+^ signals in response to mannitol. **A** Time course of changes in [Ca^2+^]_cyt_ induced by 250 mM mannitol in leaf tissue of barley and Arabidopsis. The dashed lines represent the time point of injection of the treatment. **B** Maximal increases in Δ[Ca^2+^]_cyt_ in leaf and root tissue induced by different concentrations of mannitol. **C** Time courses of changes in [Ca^2+^]_cyt_ induced by 250 mM mannitol in root tissues . The dashed lines represent the time point of injection of the treatment. **D** Time to reach the maximal increase of [Ca^2+^]_cyt_ after injection of 250 mM mannitol in seconds (s). Values represent means ± SE of three independent replicates

Mannitol is an osmotic substance commonly used to mimic drought stress [16, 36, 37]. Time-course analysis of cytosolic Ca^2+^ responses showed that barley leaf and root tips were little affected by mannitol (Fig. S4 A, B). In leaf tips, only stimulation with 250 mM mannitol caused a very small long-lasting [Ca^2+^]_cyt_ elevation (Fig. 4A; Fig. S4A). In the root tip, a fast [Ca^2+^]_cyt_ transient was observed at higher mannitol concentrations (Fig. S4B), however, even with 250 mM mannitol the [Ca^2+^]_cyt_ amplitude was less than 0.1 μM Δ[Ca^2+^]_cyt_ (Fig. 4B, C). This fast but relatively weak [Ca^2+^]_cyt_ increase lasted for 30-40 s before declining to a new, slightly higher resting level (Fig. 4C). Luminescence imaging experiments confirmed that the Ca^2+^-dependent photon release upon mannitol treatment originated only in the root system (Fig. S5).

Similar to barley, Arabidopsis showed no fast [Ca^2+^]_cyt_ increase in response to mannitol in leaves (Fig. 4A; Fig. S4C). However, in contrast to barley, mannitol induced a strong and dose-dependent response with different dynamics in Arabidopsis roots (Fig. 4B, C; Fig. S4D). Application of 250 mM mannitol induced an increase in Arabidopsis roots with a peak of about 0.5 μM Δ[Ca^2+^]_cyt_ (Fig. 4B, C). The time to reach the maximal [Ca^2+^]_cyt_ in the roots upon application of 250 mM mannitol was not significantly different between barley and Arabidopsis at around 8 to 11 s (Fig. 4D). In addition to this first spike, Arabidopsis roots showed smaller, secondary [Ca^2+^]_cyt_ elevations before returning to the basal level, which were not recorded in barley. Taken together, the results indicated that osmotically triggered Ca^2+^ signals differ between Arabidopsis and barley.

### [Ca^2+^]_cyt_ changes in barley and Arabidopsis induced by flg22

**Fig. 5:**
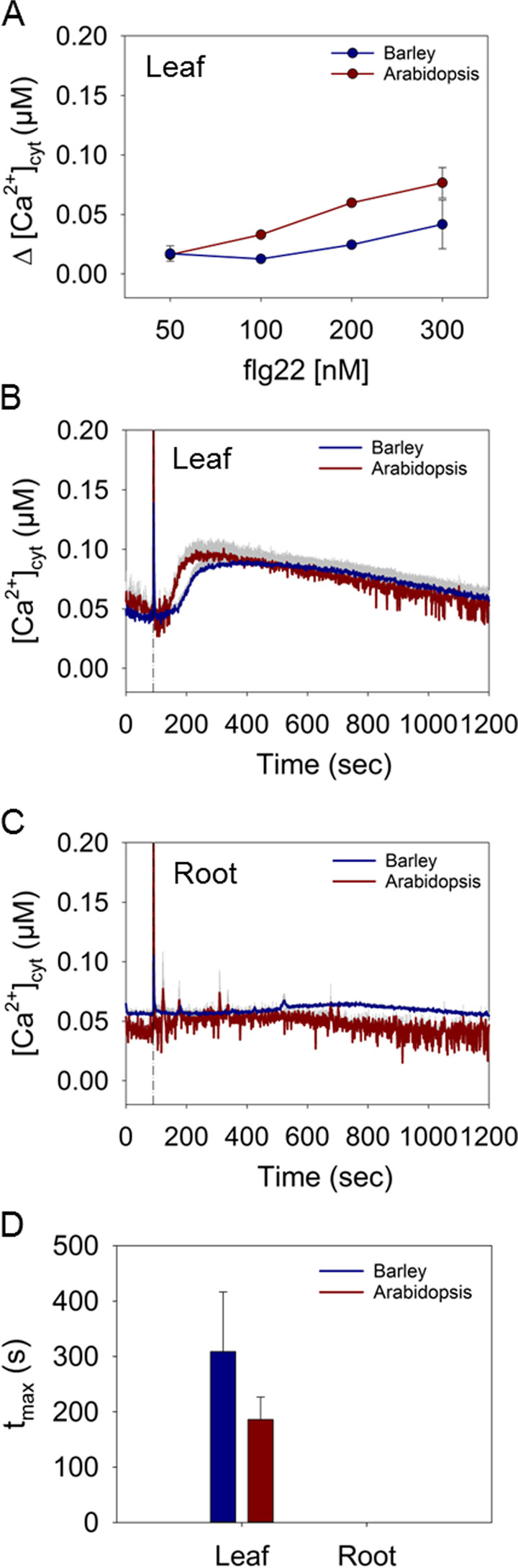
Induction of Ca^2+^ signals in response to flg22. **A** Maximal increases in Δ[Ca^2+^]_cyt_ in barley and Arabidopsis leaf tissue induced by different concentrations of flg22. Time courses of changes in [Ca^2+^]_cyt_ induced by 200 nM flg22 in leaf (**B**) and root (**C**) tissues. The dashed lines represent the time point of injection of the treatment. **D** Time to reach the maximal increase of [Ca^2+^]_cyt_ after injection of 200 nM flg22 in seconds (s). Values represent means ± SE of three independent replicates

As a biotic stimulus, effects of the PAMP flg22 on [Ca^2+^]_cyt_ transients were investigated. In barley, application of flg22 evoked a clear response only in the leaf but not in the root (Fig. 5A-C; Fig. S6A, B). The [Ca^2+^]_cyt_ elevations were induced at concentrations above 200 nM flg22 and they were relatively weak with no sharp spike (Fig. 5A, B). Treatment with 200 nM flg22 induced a Ca^2+^ transient with a maximum of 0.05 μM Δ [Ca^2+^]_cyt_ (Fig 5A, B). The increase in [Ca^2+^]_cyt_ started with a delay of approximately 90 s after injection of the elicitor and declined very slowly over minutes to the basal level (Fig. 5B). Also in Arabidopsis, a clear response was observed in leaves (Fig. 5 B; Fig. S6C). Durations and shape of the response curves were similar to barley; however, the onset of the response occurred slightly faster (Fig. 5B). Arabidopsis leaves responded to flg22 already at 100 nM, a concentration for which no signal could be recorded in barley (Fig. 5A; Fig. S6A, C). The maximal increase of [Ca^2+^]_cyt_ after application of 200 nM flg22 was reached earlier in Arabidopsis as in barley within approximately 190 s and 300 s, respectively (Fig. 5B, D). As with barley, Ca^2+^ signals in response to flg22 were not detected in the roots of Arabidopsis (Fig. 5C; Fig. S6D). This has already been described for Arabidopsis [38], and our data extend the tissue specificity of the flg22-induced Ca^2+^ response to barley.

### [Ca^2+^]_cyt_ changes in barley and Arabidopsis induced by H_2_O_2_

**Fig. 6:**
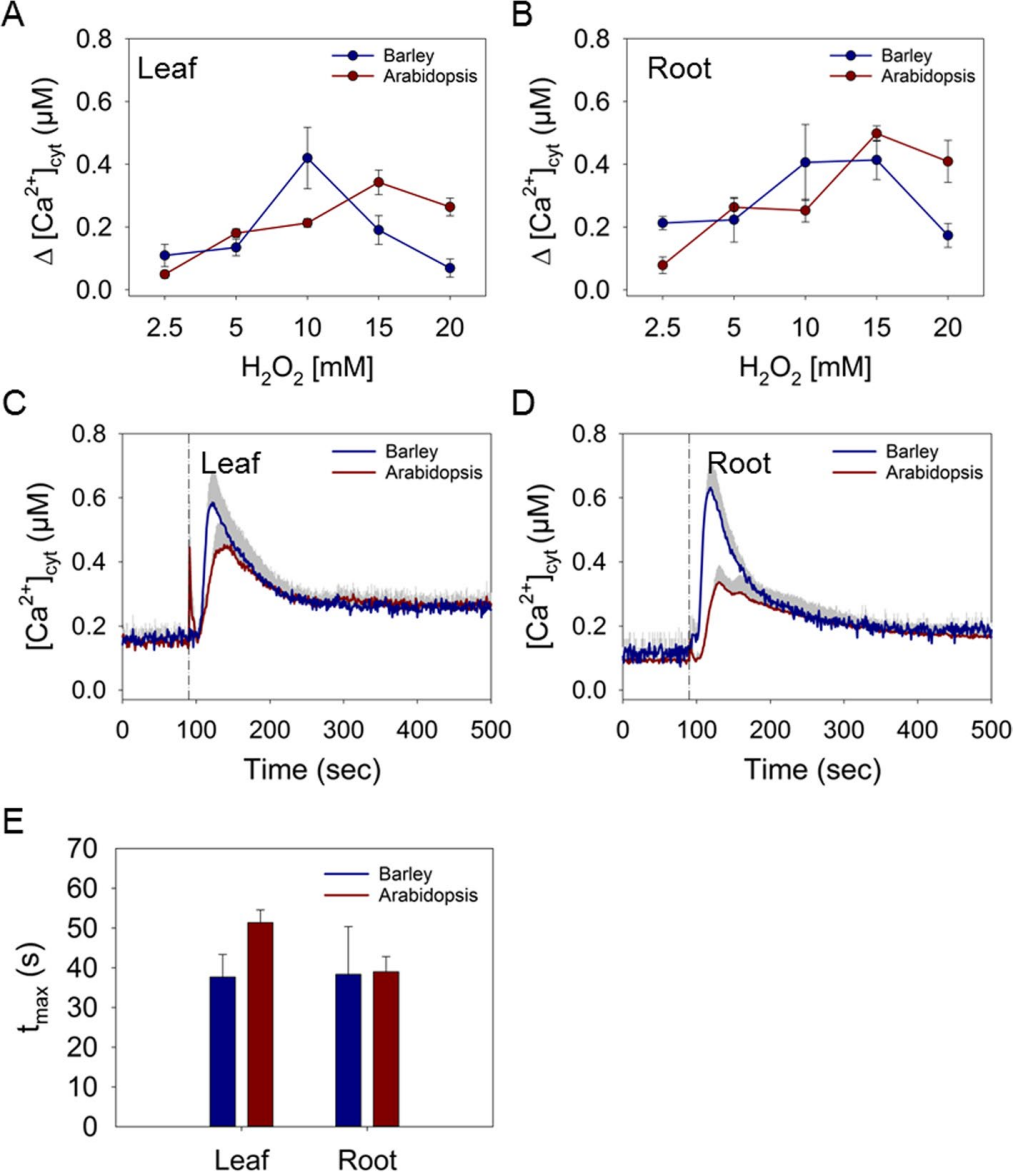
Induction of Ca^2+^ signals in response to H_2_O_2_. Maximal increases in Δ[Ca^2+^]_cyt_ in barley and Arabidopsis leaf (**A**) and root (**B**) tissue induced by different concentrations of H_2_O_2_. Time courses of changes in [Ca^2+^]_cyt_ induced by 10 mM H_2_O_2_ in leaf (**C**) and root (**D**) tissues. The dashed lines represent the time point of injection of the treatment. (**E**) Time to reach the maximal increase of [Ca^2+^]_cyt_ after injection of 10 mM H_2_O_2_ in seconds (s). Values represent means ± SE of three independent replicates

Ca^2+^ dynamics in response to oxidative stress were analysed by treatment with the reactive oxygen species H_2_O_2_. Analysis of the [Ca^2+^]_cyt_ responses showed that in barley H_2_O_2_ induced a dose-dependent response in form of a sharp albeit slightly delayed increase in [Ca^2+^]_cyt_ in both tissues (Fig. 6A – D; Fig. S7A, B). The maximal Δ[Ca^2+^]_cyt_ increase of about 0.4 μM was reached at a concentration of 10 mM H_2_O_2_, while application of higher concentrations of H_2_O_2_ resulted in a reduced response (Fig. 6A, B). Instead of a fast return to the baseline, the [Ca^2+^]_cyt_ declined gradually within 100 s to a new and elevated level (Fig. 6C, D; Fig. S7A, B). Treatment of Arabidopsis leaves and roots with 10 mM H_2_O_2_ resulted in [Ca^2+^]_cyt_ increases much lower than those induced in barley with a peak at about 0.2 and 0.25 μM Δ[Ca^2+^]_cyt_ in leaves and roots (Fig. 6 A-D; Fig. S7C, D). The response in Arabidopsis could be further elevated by higher H_2_O_2_ concentrations, reaching peak heights similar to barley at 15 mM and also decreasing upon higher concentrations (Fig. 6A, B; Fig. S7C, D). In both species, the maximal increase in [Ca2+]_cyt_ upon treatment with 10 mM H_2_O_2_ was reached within approximately 40 s, with the exception of Arabidopsis shoots that responded a little slower at about 50 s (Fig. 6E). Also, the shape of the [Ca^2+^]_cyt_ transients in Arabidopsis differed from those in barley (Fig. S7), with a broader peak and an additional secondary shoulder, indicating differences in H_2_O_2_-triggered Ca^2+^ signalling in both species.

### Spatial distribution of stimulus-induced [Ca^2+^]_cyt_ responses in barley leaves

**Fig. 7:**
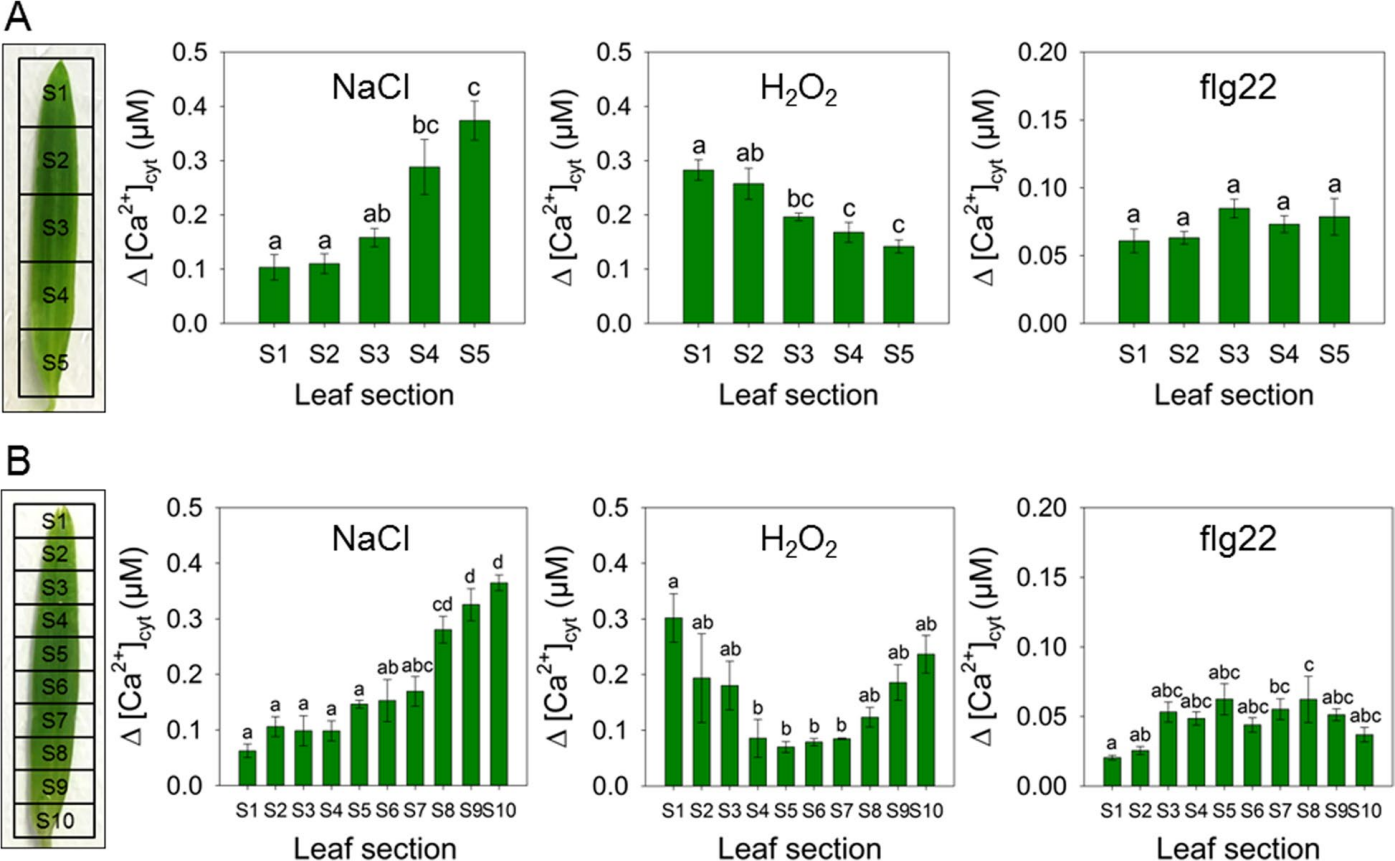
Changes in [Ca^2+^]_cyt_ in response to NaCl, H_2_O_2_, and flg22 in different sections of barley leaves. Maximal increases in [Ca^2+^]_cyt_ in different leaf sections, as shown in the left panel, of five-day-old (**A**) or seven-day-old (**B**) barley plants in response to 250 mM NaCl, 10 mM H_2_O_2_, and 200 nM flg22. Pictures of the partitioning of the barley leaves are representative. Values represent means ± SE of three independent replicates. Letters represent significant differences between the sections according to a One-way ANOVA with Tukey test (*P*< 0.05)

In the experiments described above, we measured [Ca^2+^]_cyt_ in the leaf tip of barley. However, in barley, growing leaves show a developmental gradient along the leaf blade [39, 40]. To investigate whether the stimulus-induced [Ca^2+^]_cyt_ response is uniform along this developmental gradient, we analysed spatio-temporal dynamics of the [Ca^2+^]_cyt_ transients in different sections of the leaf. For this, leaves of five- and seven-day-old Hv-AEQ_cyt_#18 plants, were separated into 5 mm sections from the tip to the base resulting in five and ten parts, respectively (Fig. 7). The sections were challenged with 250 mM NaCl, 10 mM H_2_O_2_ or 200 nM flg22. Mannitol was not tested since it did not show any [Ca^2+^]_cyt_ response in the leaf tip. In all leaf sections, there were little temporal differences in [Ca^2+^]_cyt_ increases upon application of the stimuli (Fig. S8). However, the peak heights showed a specific spatial distribution along the leaf blade in response to NaCl and H_2_O_2_ (Fig. 7; Fig. S8).

In the five-day-old leaves, the intensity of the Ca^2+^ signal increased in a linear gradient from the tip (S1) to the base (S5) in case of NaCl application (Fig. 7A), while the H_2_O_2_-treated leaves showed the strongest response in the tip, which decreased towards the base (Fig. 7A). Barley leaves grow at the base of the leaf; therefore, the topmost five sections of the seven-day-old leaves corresponded to the five sections of the five-day-old leaves, with the additional five sections representing newly grown tissue. Similar to five-day-old plants, the response to NaCl increased from the top towards the leaf base also in seven-day-old barley plants. However, whereas the response to H_2_O_2_ in the five upmost sections of a seven-day-old plant mirrored the decline observed in five-day-old plants, the response increased again progressively in the last sections toward the basal part of the leaf (Fig. 7B). Whereas no significant differences in the intensity of [Ca^2+^]_cyt_ along the leaf blade were recorded in five-day-old plants treated with flg22, the amplitude of the peaks were slightly increased in the elongation zone of seven-day-old plants (Fig 7). To confirm that the physical wounding of the leaves in consequence of the sectioning did not alter the stress response, aequorin-based luminescence signals were analysed in five-day-old intact plants in response to H_2_O_2_ using a photon-counting camera system (Fig. S9A). Indeed, similar response patterns to the sectioned leaf were observed with the leaf tip showing the highest Ca^2+^ signal and a decreasing trend along the blade (Fig. S9B, C).

### Contribution of external Ca^2+^ stores to stress-induced [Ca^2+^]_cyt_ transients in barley

**Fig 8:**
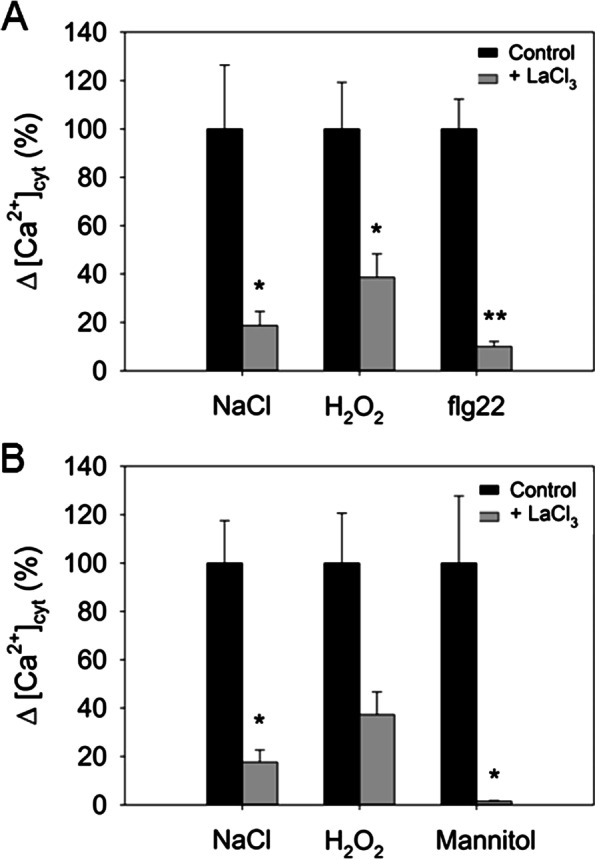
Effect of LaCl_3_ pre-treatment on stress-mediated Ca^2+^ signals. Inhibitory effect on the maximal [Ca^2+^]_cyt_ increase in response to 200 mM NaCl, 10 mM H_2_O_2_, 200 nM flg22, and 200 mM mannitol after pre-treatment of barley leaf tips (**A**) and root tips (**B**) with 1 mM LaCl_3_ for one hour relative to control conditions without pre-treatment. Values represent means ± SE of three independent replicates. Asterisks indicate significant differences from the control without pre-treatment according to Student’s *t* test (* = *P*>0.05; ** = *P*>0.01)

La^3+^, a widely used blocker of plasma membrane Ca^2+^ channels [34] was used to investigate the contribution of the apoplast to the [Ca^2+^]_cyt_ elevations in barley (Fig. 8). Pre-incubation of samples with LaCl_3_ had similar inhibitory effects on [Ca^2+^]_cyt_ responses to NaCl in both leaf and root tips with about 80% inhibition (Fig. 8). In the case of H_2_O_2_, the effect of LaCl_3_ was slightly weaker in both tissues with about 60% inhibition. For mannitol and flg22, only root tips or leaf tips were analysed, respectively, because the response to these stimuli was strictly tissue-specific (Fig. 4, 5). Here, LaCl_3_ resulted in a 90% inhibition of the response to flg22 in the leaf tips and almost complete inhibition of the response to mannitol in root tips. Overall, these results demonstrate the requirement of Ca^2+^ influx from the apoplast for the generation of the response to all tested stimuli in both leaves and roots but also suggests a minor role of internal stores.

## Discussion

The aequorin bioluminescence reporter system is a powerful tool to visualize [Ca^2+^] transients and thus to analyse Ca^2+^ signals that occur in response to a variety of biotic and abiotic stresses *in planta* [12, 41]. In this work, we established barley lines harbouring aequorin in the cytosol. The transgenic lines show a Ca^2+^-dependent photon release after discharge, stimulus-dependent [Ca^2+^]_cyt_ transients, and no discernible phenotypic differences to the wild type (Fig. 1, 2; Fig. S2). Therefore, these lines were suitable to analyse Ca^2+^ signals in response to different stimuli in the crop species barley and to compare them with those in the model plant Arabidopsis.

Overall, barley and Arabidopsis showed stimulus-induced [Ca^2+^]_cyt_ responses with similar tissue-specificity. Both plants reacted strongly to NaCl and H_2_O_2_ in leaves as well as in roots. By contrast, the response to mannitol and flg22 was tissue-dependent, taking place either in the root or in the leaf, respectively (Figs. 3, 4, 5 and 6). Main differences between barley and Arabidopsis occurred with regard to amplitude and shape of the response curve as well as the intensities of the stimuli required to elicit an increase in [Ca^2+^]_cyt_. These disparities might be indicators for species-specific differences in stress response mechanisms.

The [Ca^2+^]_cyt_ transients differed in osmotic stresses triggered either by salinity (ionic) (Fig. 3) or mannitol (non-ionic) (Fig. 4). In both, Arabidopsis and barley, a dose-dependent [Ca^2+^]_cyt_ response to NaCl occurred in form of an initial sharp spike, sometimes followed by several much smaller spikes (Fig. 3; Fig. S3). The latter might be due to other factors inducing a secondary response, with H_2_O_2_ being a likely candidate [42]. For Arabidopsis, it has also been suggested that these secondary Ca^2+^ spikes might arise from different cell populations within the tissues [33, 34]. While the maximum height of the [Ca^2+^]_cyt_ increase in the root was similar between the two plants, the Ca^2+^ response in shoot was higher in the more salt-sensitive Arabidopsis. In Arabidopsis, the SOS (salt overly sensitive) pathway has been suggested to be essential for salt tolerance [43]. The SOS pathway consists of a Ca^2+^ sensor-protein kinase complex (SOS3/CBL4-SOS2/CIPK24), which activates the Na^+^/H^+^ antiporter SOS1 by phosphorylation to clear cytosolic Na^+^ during salt stress. The higher responsiveness of Arabidopsis shoots thus might be related to an increased Na^+^ accumulation due to an inferior ability of this plant to exclude Na^+^. Components of the SOS pathway are conserved in several plants including crops like maize, rice, and tomato [44–47], but have not yet been characterized in barley.

By contrast to salt, osmotic stress triggered by mannitol mimics drought stress and caused transient and dose-dependent increases in [Ca^2+^]_cyt_ in the roots of barley and Arabidopsis, whereas leaves of both species did not show any response (Fig. 4; Fig. S4 and 5). Similar to NaCl, both plants showed a rapid initial [Ca^2+^]_cyt_ increase in response to mannitol; however, the response in barley roots was much less pronounced compared to Arabidopsis (Fig. 4C). Furthermore, barley root tips showed a prolonged [Ca^2+^]_cyt_ elevation slightly above the baseline level, while Arabidopsis showed several secondary peaks but no sustained elevation (Fig. S4). As with salt, barley is considered to have a higher tolerance to this abiotic stress [48], and this might be related to the different [Ca^2+^]_cyt_ transients observed. Due to the concomitantly reduced diffusion of K^+^ in dry soils, drought stress is known to impair the influx of K^+^ across the plasma membrane [49]. However, K^+^ serves as a compatible osmolyte under osmotic stress. Hence, in barley mannitol was shown to initiate K^+^ fluxes for initial osmotic adjustment likely mediated by activation of inward-rectifying K^+^ channels and transporters in the plasma membrane [50]. The primary root-borne K^+^ uptake systems of Arabidopsis, such as AKT1 and HAK5, are activated by Ca^2+^-mediated phosphorylation [51] and may hence present a target of the osmotically induced Ca^2+^ signal. The consequence of the lower amplitude of the signal in barley on the activation of K^+^ fluxes remains to be elucidated.

In Arabidopsis, Ca^2+^ elevations in response to hyperosmotic stress depend on OSCA1, which operates as an osmosensor in the root and guard cell plasma membrane [52, 53]. In addition, Ca^2+^ channel-forming annexins activated by hydroxyl radicals may play a role under osmotic and salt stress conditions [54], a mechanism that has also been suggested for barley [50]. OSCAs and annexins are likely candidates for the generation of Ca^2+^ signals in this crop, yet this remains to be shown. Salinity and hyperosmotic stress activate in addition phosphoinositide signalling that leads to the generation of two secondary messengers, inositol 1,4,5-trisphosphate (IP_3_) and 1,2-diacylglycerol [55, 56], required for the accumulation of proline in Arabidopsis and barley [57, 58]. In animals, IP_3_ acts as a Ca^2+^-mobilizing ligand of IP_3_ receptor Ca^2+^ channels in endomembranes. Although early biochemical and electrophysiological studies pointed to a similar mechanism in plants, there is no genetic evidence for this [59].

Contrary to mannitol, flg22-induced [Ca^2+^]_cyt_ transients were mainly observed in leaves in both species (Fig. 4; Fig. S6). Though flg22 responses have been shown in Arabidopsis shoots and roots by Beck and co-workers [60], our results are in agreement with other studies showing only marginal changes in [Ca^2+^]_cyt_ in Arabidopsis roots upon flg22 treatment [61]. Time-course analysis showed a long-lasting response in barley leaf tips with kinetics comparable to those in Arabidopsis shoots. In Arabidopsis, this aequorin-based read-out of [Ca^2+^]_cyt_ transients triggered by flg22 is an integration of Ca^2+^ oscillations in non-synchronized individual cells, as shown for guard cells, which provoke stomatal closure to prevent pathogen entry [14]. It is likely but remains to be demonstrated that flg22 triggers an oscillatory response also in barley. The most obvious difference between Arabidopsis and barley was the higher concentration of flg22 required to elicit a response in barley shoots (Fig. S6). This may be caused by different affinity of the flg22 sensor, FLS2, in both species and may translate to different thresholds of defence responses. While an increases in [Ca^2+^]_cyt_ is known to be crucial for cellular downstream responses [61] many of the signalling events downstream of flg22 sensing have been elucidated in Arabidopsis, but not yet analysed in barley. This includes the mechanism of flg22-triggered Ca^2+^ signal generation by a plasma membrane-localized Ca^2+^ channel, OSCA1.3, which mediates the guard cell-specific Ca^2+^ influx upon phosphorylation by the cytosolic immune receptor-associated protein kinase BIK1 [62]. The operation of OSCA1.3 orthologs in barley remains to be shown. In Arabidopsis, flg22-induced [Ca^2+^]_cyt_ signals partially depend on an oxidative burst, generated by NADPH oxidases [14]. Thereby NADPH/respiratory burst oxidase D (RBOHD) and, to a lesser extent, RBOHF, generate ROS in the apoplast [63]. Interestingly, in barley the flg22-induced oxidative burst was independent from RBOHF orthologs suggesting that, compared to Arabidopsis, yet unidentified players are involved [64].

H_2_O_2_ was used to mimic the effect of oxidative stress caused by ROS generated inside the cell or in the apoplast upon other stresses such as biotic attack, salt stress, or impairment of photosynthesis [65]. However, ROS are also formed by the cell as intercellular messengers, e.g. as a response to other stresses, and even contribute to systemic Ca^2+^ signal propagation [66]. Application of H_2_O_2_ resulted in [Ca^2+^]_cyt_ transients in barley and Arabidopsis, both in roots and leaves (Fig. 6; Fig. S7). Compared to salt and drought stress, the response to H_2_O_2_ appeared 15-20 seconds later and showed an extended duration of the [Ca^2+^]_cyt_ transient for several minutes, often not returning to the baseline within the measurement period. It is known that ROS and Ca^2+^ signalling interact with each other in a way that elevation in [Ca^2+^]_cyt_ induces ROS production and *vice versa,* thus extending the duration and amplitude of the signals [67, 68]. In the case of H_2_O_2_, a clear difference in the concentration dependency of the response could be observed between barley and Arabidopsis. This may indicate a higher sensitivity or responsiveness of barley to oxidative stress compared to Arabidopsis. However, the [Ca^2+^]_cyt_ responses require that H_2_O_2_ penetrates the tissue, and it cannot be excluded that this is a decisive factor for the observed differences.

A growing barley leaf represents successive developmental zones with functionally and developmentally distinct cells: the mature zone at the tip with fully differentiated mature cells, the division zone at the base of the blade with dividing cells, and in between the elongation zone with expanding cells [39, 40]. Ca^2+^ signals analysed in different sections of leaves from five- and seven-day-old Hv-AEQ_cyt_ #18plants after application of NaCl, H_2_O_2_, or flg22 showed that there are quantitative differences in Ca^2+^ responses (Fig. 7; Fig. S8), implying a relevance of the developmental stage of the cells in the response to these stimuli. In response to NaCl, the strongest signal was detected at the base of the diffusion zone which then decreased progressively through the elongation to the mature zone, reaching the lowest intensity in the leaf tip. Conversely, in response to H_2_O_2_, the strongest increase in [Ca^2+^]_cyt_ was obtained at the tip of the leaf of five-day-old plants, whereas in seven-day-old leaves, the strongest signals were obtained not only in the leaf tip but also at the base and decreased towards the centre from both regions (Fig. 7). The intensity of the signal in response to flg22 showed marginal variation along the leaf blade with only a slightly higher intensity in the middle zone of leaves of seven-day-old plants (Fig. 7). The observed differences provide evidence that the early stages of the Ca^2+^ signalling pathway in response to NaCl and H_2_O_2_ might differ in a developmental-stage dependent manner in barley leaves. This raises the question whether there is a degree of specificity in the Ca^2+^ signalling pathway in leaves related to the developmental stage of the tissues. Such cell type-specific Ca^2+^ patterns have also been observed in Arabidopsis, but so far only in roots [33]. Moreover, the quantitative differences in Ca^2+^ responses in barley may be due to a cell type-specific recruitment of the components involved upstream of the Ca^2+^ signal, such as stimuli sensors, signal transmission molecules, Ca^2+^ influx channels, and Ca^2+^ exporters, that are poorly understood in Arabidopsis and yet unidentified in barley.

La^3+^ has been widely used as a blocker of Ca^2+^ channels located at the plasma membrane [14, 69]. This inhibitor reduced all stress-induced [Ca^2+^]_cyt_ transients, albeit with varying effectiveness (Fig. 8). In the most extreme case, La^3+^ almost completely abolished the [Ca^2+^]_cyt_ response to mannitol in the root tips. These results unequivocally demonstrate that Ca^2+^ influx from the apoplast is crucially involved in the generation of Ca^2+^ signals in response to all examined stresses in barley. However, La^3+^ did not abolish the response completely, pointing to the additional involvement of internal Ca^2+^ stores. Such a co-operation of Ca^2+^ stores is in line with many studies on Arabidopsis and rice [14, 16, 31]. The different Ca^2+^ sources may act independently or in the same pathway. Possible mode of actions may consist in feed-forward loops, like Ca^2+^-induced Ca^2+^ release [21]. Such a signal amplification may be mediated by [Ca^2+^]_cyt_-activated Ca^2+^-permeable channels, like TPC1 [70].

## Conclusions

Overall, this study showed that the bioluminescent Ca^2+^ reporter aequorin is a powerful tool to measure [Ca^2+^]_cyt_ dynamics in barley. It further showed that Ca^2+^ signalling is involved in the early stages of responses to environmental cues in this species and that the Ca^2+^ signatures in response to NaCl, H_2_O_2_, mannitol, and flg22 are quite comparable to those in Arabidopsis. However, it also revealed notable differences in temporal kinetics and intensity of stress-mediated [Ca^2+^]_cyt_ elevations. Moreover, differences in the concentration of stimuli required to induce such signals, have been observed. Such disparities between barley and Arabidopsis suggest a species specificity in Ca^2+^-dependent stress response mechanisms and may be indicative for the involvement of different molecular machineries in the generation of Ca^2+^ responses. Therefore, future investigations are necessary to identify the molecular identity of the components of these machineries. In that context, it is of great advantage that the genomic sequence of barley is available [71], enabling genetic approaches to investigate the components of the Ca^2+^ signalling toolbox in barley. The barley *APOAEQUORIN* lines described here can further assist in these investigations.

## Methods

### Vector construction and transformation of barley

For the generation of barley aequorin lines (Hv-AEQ_cyt_) the coding region of *APOAEQUORIN* was amplified from the vector pBIN19-AEQ [12] using the forward primer 5´-ATGACCAGCGAACAATACTCAGTC-3´ and the reverse primer 5´- CGGTGGAGCTGTCCCCTAA-3´ containing XmaI restriction sites and cloned upstream of *Zea mays ubiquitin-1* promoter (*ZmUBI1*) into the vector pUbi-AB (DNA Cloning Service, Hamburg, Germany [72]). The entire expression cassette was sub-cloned into the binary vector pLH7000 (DNA Cloning Service) via the SfiI restriction site. This construct was introduced in *Agrobacterium tumefaciens* strain AGL-1 [73] through electroporation (Gene Pulser Xcell Electroporation Systems; Bio-Rad). Agrobacterium-mediated gene transfer was performed with the barley wild type cultivar Golden Promise as described [74]. To confirm the functionality of aequorin, the luminescence of Hv-AEQ_cyt_ lines was analysed by discharging aequorin with 1 M CaCl_2_ in 10% ethanol in a plate luminometer (Mithras LB940, Berthold Technologies, Pforzheim, Germany) as described below.

### Plant material and growth conditions

Transgenic Hv-AEQ_cyt_ barley plants (T3) homozygous for *APOAEQUORIN* as well as plants of a transgenic Arabidopsis Col-0 line expressing cytosolic *APOAEQUORIN* [12] were grown in pots filled with water-soaked vermiculite. Plants were cultivated under long-day conditions (16 h light, 20°C with a light intensity of 100-120 μmol m^-2^ s^-1^ and 8 h darkness, 18 °C; 65% rh) in climate-controlled growth cabinets. The *APOAEQUORIN* sequences in Arabidopsis and barley are identical.

### Quantification of *APOAEQUORIN* transcript levels and aequorin immunodetection

For quantification of *APOAEQUORIN* transcript levels, mRNA of seven-day-old Hv-AEQ_cyt_ seedlings was isolated from leaves and roots using a Spectrum Plant Total RNA kit (Sigma-Aldrich, St. Louis, MO, United States) with on-column DNase I (Omega Bio-Tek, Norcross, GA, USA) treatment according to the manufacturer´s instructions. cDNA was synthesized from 1 μg of mRNA using random hexamer primers and M-MLV reverse transcriptase (Promega, Madison, WI, USA) according to the manufacturer´s protocol. Quantitative real-time PCR was carried out in a realplex^4^ MasterCycler system (Eppendorf, Hamburg, Germany) using the Power SYBR Green PCR master mix (Applied Biosystems, Foster City, CA, USA). For amplification of 120 bp of *APOAEQUORIN*, forward primer 5´-CAAGGCGTCCGATATTGTTATAAA-3´ and reverse primer 5´-TGGAATGAAATATGGTGTAGAAACTGA-3´ were used. The expression level was quantified using a cDNA dilution series and normalized to actin *HvACTIN2* (AY145451.1) as constitutively expressed control.

For immunodetection of APOAEQUORIN, proteins were isolated from leaves and roots of five-day-old barley wild type and Hv-AEQ_cyt_ plants using a protein extraction buffer as previously described [75]. The proteins were separated on a 12% SDS-polyacrylamide gel and transferred to PVDF membrane (Thermo Scientific, Waltham, MA, USA). Immunodetection was performed using an antibody against aequorin (Abcam, Berlin, Germany) and an ECL detection system (Serva, Heidelberg, Germany) with an anti-rabbit secondary antibody coupled to horseradish peroxidase (Sigma-Aldrich).

### Phenotypic analyses

Thousand grain weight was determined by weighing 100 grains of 4 independent plants of Hv-AEQ_cyt_ #18 and the wild type in duplicates and extrapolating by the factor of 10. Fresh weight, root length, and leaf blade length were determined of eight individual seedlings grown for three or five days on vermiculite under the same growth conditions as used for the experiments.

### Aequorin reconstitution and luminescence measurements

Unless otherwise stated, 5 mm sections from the tip of the leaf and the primary root of three-day-old Hv-AEQ_cyt_ seedlings were used for [Ca^2+^]_cyt_ measurements in barley, while entire shoots or roots of five-day-old seedlings were used for Arabidopsis. Tissues were reconstituted for 16 hours in the dark in 2.5 μM coelenterazine solution (Carl Roth, Karlsruhe, Germany). After reconstitution, the coelenterazine solution was replaced by in ddH_2_O, and tissues were allowed to recover for one hour in light before measurements. For inhibitor treatments, leaf or root tips of barley were incubated for one hour in 1 mM lanthanum chloride (LaCl_3_) in ddH_2_O after reconstitution. All measurements were performed in 96-well plates (Lumitrac 600, Greiner Bio-One, Kremsmünster, Austria) in a plate luminometer (Mithras LB940). Luminescence was detected for 90 seconds with an integration time of 1 sec to record the baseline before the injection of an equal volume of a 2-fold-concentrated solution of H_2_O_2_, NaCl, mannitol, or flg22. After injection, changes in luminescence were recorded for another 600 sec (H_2_O_2_, NaCl, mannitol) or 1200 sec (flg22). After injection of discharge solution (final concentration: 1 M CaCl_2_ in 10% ethanol) luminescence was recorded for another 300 sec with the same integration time. [Ca^2+^]_cyt_ was calculated as described [76] with a background correction acquired from measurements of empty wells under the same conditions. To calculate Δ [Ca^2+^]_cyt_, the mean [Ca^2+^]_cyt_ derived from 10 sec of baseline prior to treatment was subtracted from the maximum [Ca^2+^]_cyt_ obtained after injection. All experiments were repeated at least twice with similar results.

For luminescence imaging, intact Hv-AEQ_cyt_ plants were mounted on a Petri dish using double faced adhesive tape and *APOAEQUORIN* was reconstituted by spraying leaves with 10 μM coelenterazine in 0.01% Tween 20 and subsequent incubation for six hours in the dark. Aequorin imaging was performed according to [15] using a high-resolution photon-counting camera system (HRPCS218; Photek, St Leonards on Sea, UK). Plants were placed in the dark box of the system, and photons were recorded in photon-counting mode with a frame rate of 200 ms. Treatment solutions were injected in the closed dark box via a tubing system 90 seconds after the beginning of the measurement. The remaining aequorin was discharged with 1 M CaCl_2_ in 10% ethanol. Ca^2+^-dependent light emission was analysed with the IFS32 software (Photek) by drawing defined regions of interest (ROIs) and normalized by calculating *L/L*_*max*_ (luminescence counts per sec / total luminescence counts remaining) for each ROI. All experiments were repeated at least twice with similar results.

#### Statistical analysis

All experiments were repeated at least twice. The data on which the graphs and bar plots in figures are based are shown in the Additional Data Files 2-5. Statistical analyses were performed using Sigma Plot 13.0 (Systat Software).

## Supplementary Information

**Additional file 1.** **Figures S1** **to Figures S9**

**Additional file 2.** **Table S1**

**Additional file 3.** **Table S2**

**Additional file 4.** **Table S3**

**Additional file 5.** **Table S4**

## Data Availability

All data generated or analysed during this study are included in this published article and its supplementary information files.

## Abbreviations

[Ca^2+^]_cyt_: cytosolic Ca^2+^ concentration
Δ[Ca^2+^]_cyt_: amplitude of the [Ca^2+^]_cyt_ increase
CBL: calcineurin B-like protein
CIPK: CBL interacting protein kinase
flg22: flagellin22
Hv-AEQ_cyt_: barley ‘Golden promise’ line carrying apoequorin
IP3: inositol 1,4,5-trisphosphate
PAMPs: pathogen-associated molecular patterns
RBOH: NADPH/respiratory burst oxidase
SOS: salt overly sensitive
