# Supplementary material for: Comparative analysis of stress-induced calcium signals in the crop species barley and the model plant Arabidopsis thaliana
Source: BMC Plant Biol. 2022 Sep 17;22:447. doi: 10.1186/s12870-022-03820-5 (PMC9482192; doi:10.1186/s12870-022-03820-5)
Supplement: Supplementary file 1 — Additional file 1. Figures S1 to Figures S9 [file 12870_2022_3820_MOESM1_ESM.pdf]

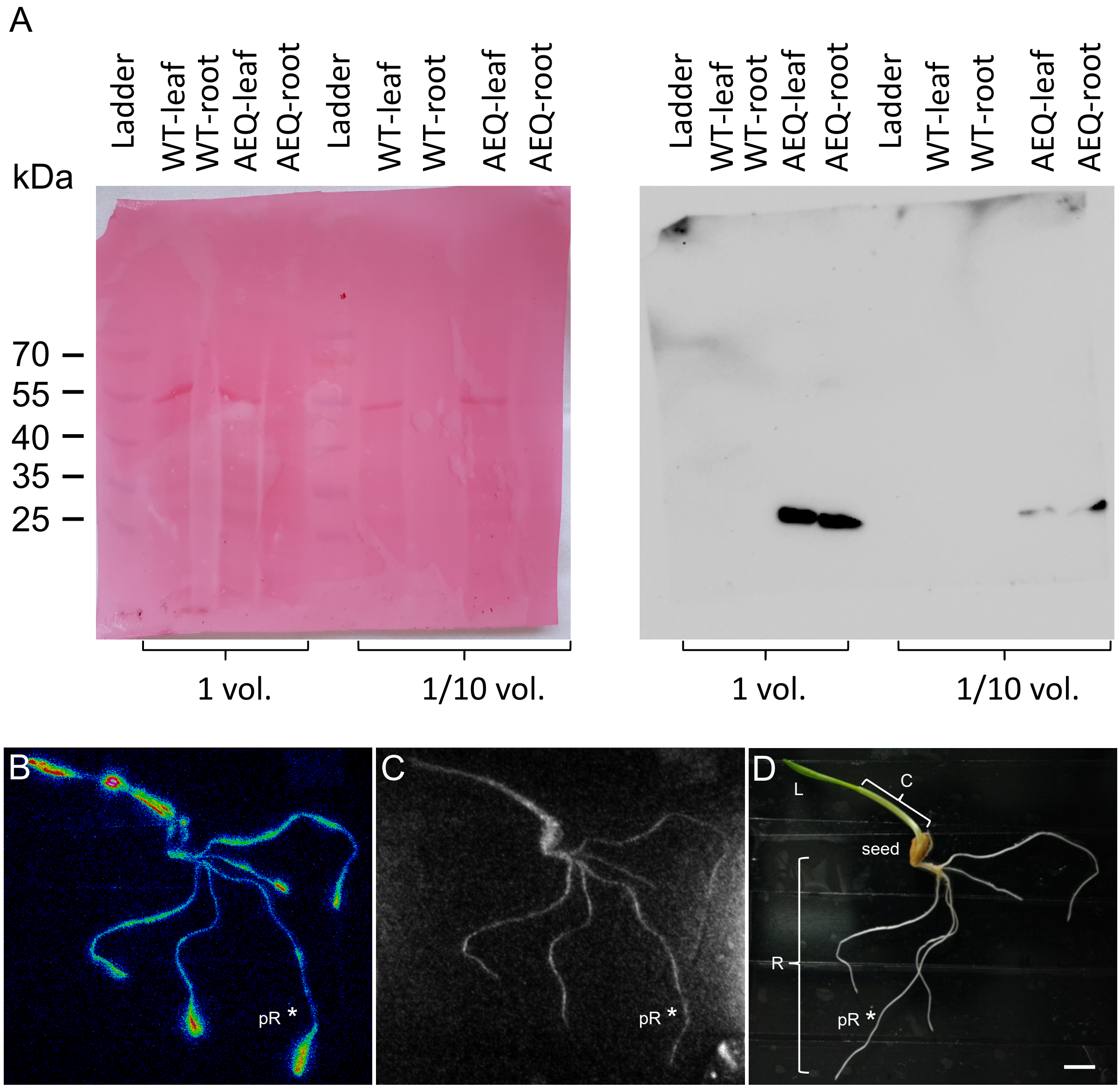

Fig. S1: Expression of aequorin and aequorin-dependent luminescence in leaves and roots of Hv-AEQ$_{cyt}$ #18. (**A**) Abundance of aequorin in leaves and roots of Hv-AEQ$_{cyt}$ #18 and wild type (WT) determined by immunodetection. Ponceau stained PVDF membrane (left) and ECL detection after Western Blot using a specific antibody against aequorin and a secondary antibody coupled to horseradish peroxidase (right). (**B**) Cumulative image of Ca^{2+}-dependent photon counts in response to a discharge of reconstituted aequorin by application of 1 M $CaCl_2$ in 10% ethanol acquired by a photon-counting camera. (**C**) Bright field image of the plant acquired by the photon-counting camera. (**D**) SLR camera image of the plant. C: coleoptile; L: emerged leaf; R: roots; pR: primary root; bar = 1 cm. Position of the primary root (labelled by *) was changed between taking the images.

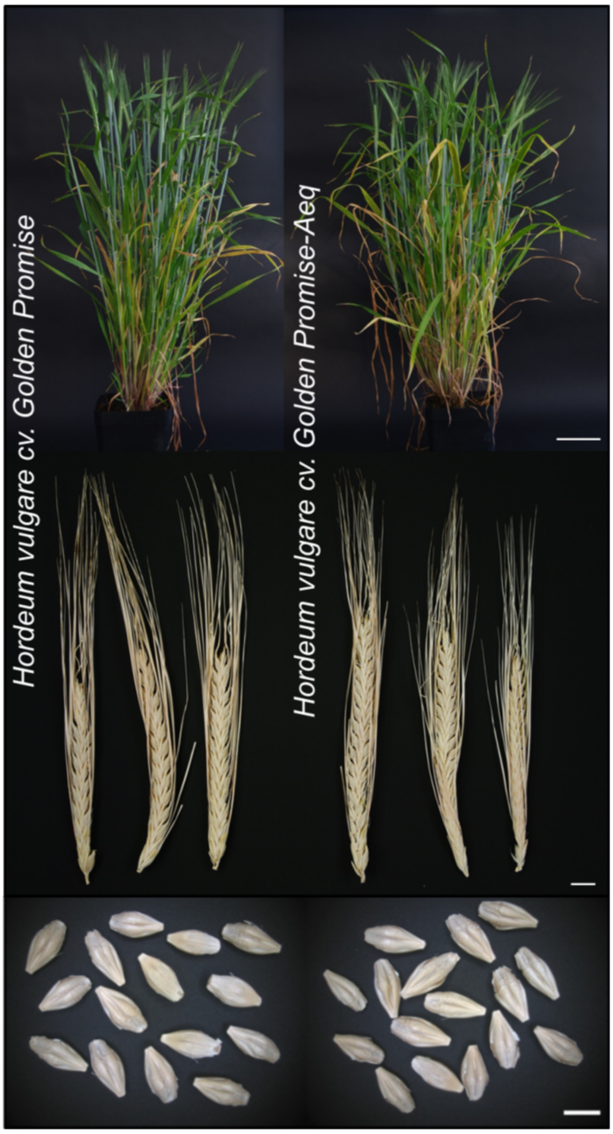

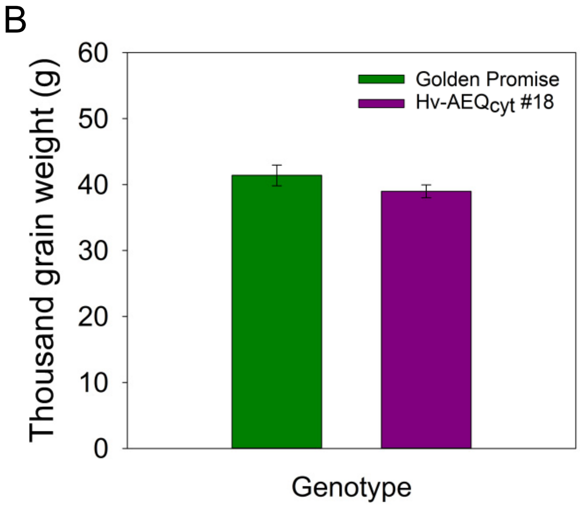

Fig. S2: Hv-AEQ$_{cyt}$ #18 shows no visible defects in development. Photographs of barley wild type plants *cv.* Golden Promise (left panel) and transgenic Hv-AEQ$_{cyt}$ #18 line (right panel) during maturation, as matured spikes, and as seeds. Bar = 10 cm (upper panel), 1 cm (middle panel), and 0.5 cm (lower panel). (**B**) Thousand grain weight.

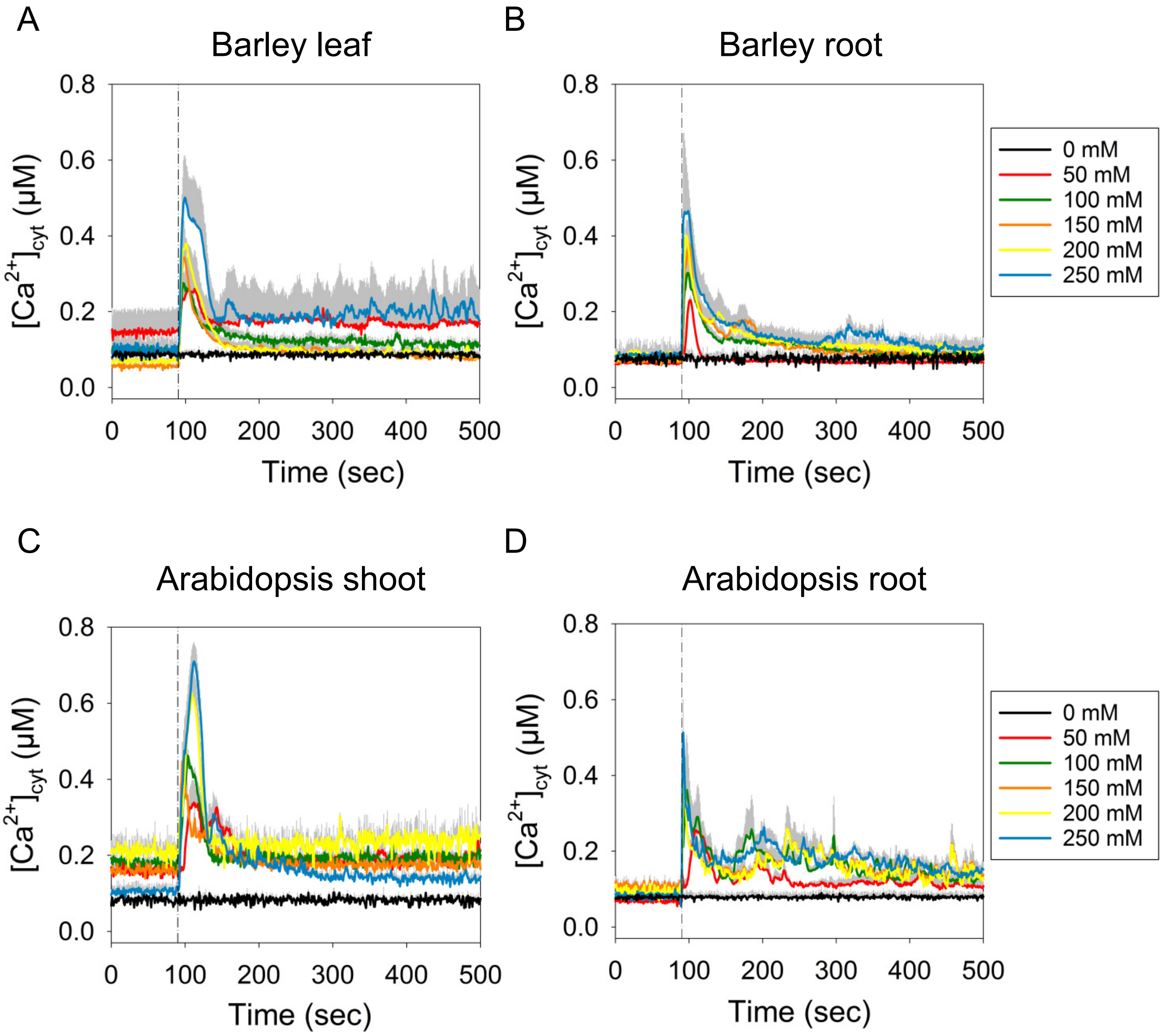

Fig. S3: Kinetics of $[Ca^{2+}]_{cyt}$ in response to different concentrations of NaCl. Time courses of $[Ca^{2+}]_{cyt}$ (µM) induced by various concentrations of NaCl in barley leaf tips (**A**), root tips (**B**), and Arabidopsis shoots (**C**) and roots (**D**) determined in a luminometer. Dashed lines represent the time point of injection of the different treatments. Values represent means ± SE of three independent replicates.

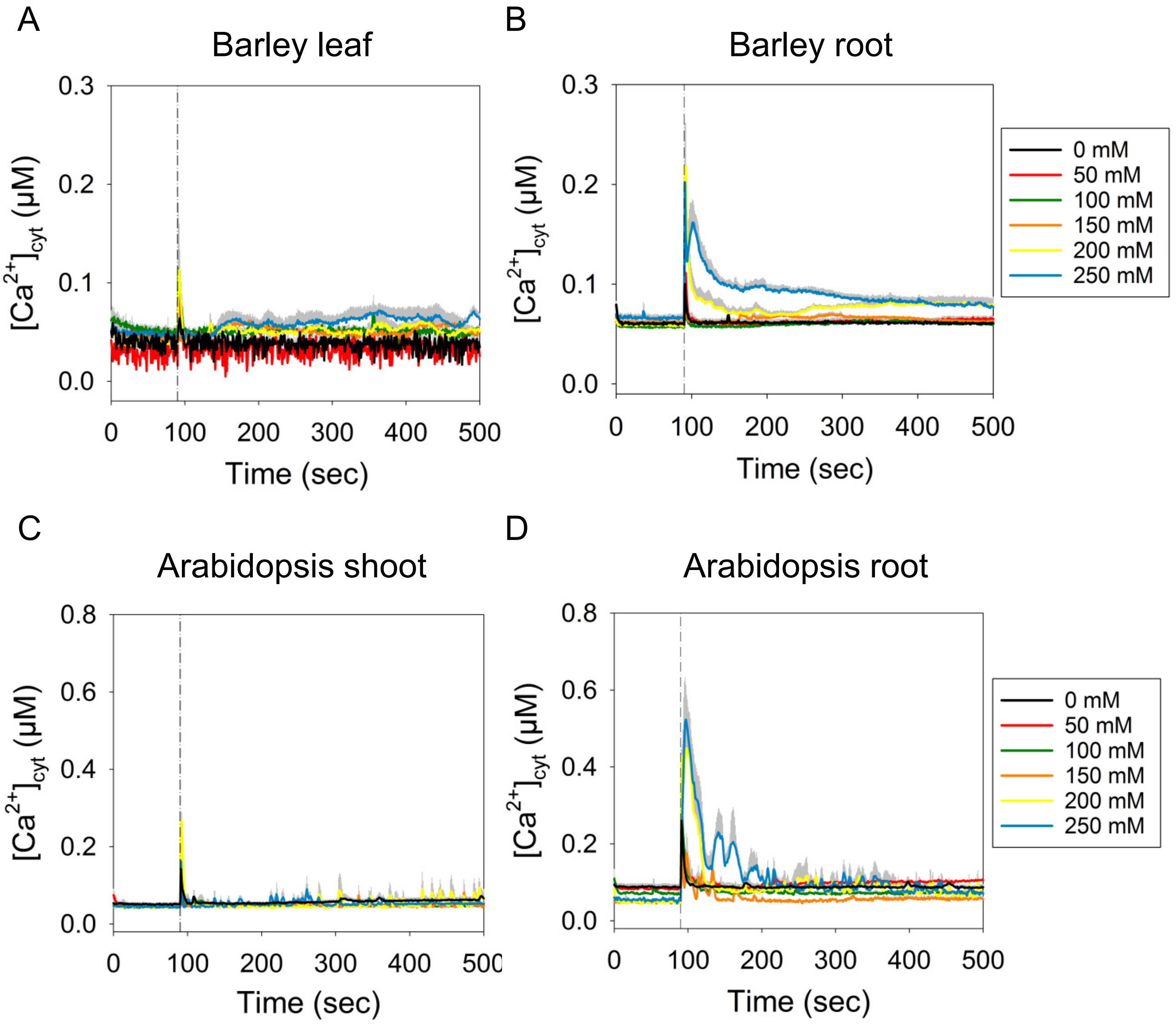

Fig. S4: Kinetics of $[Ca^{2+}]_{cyt}$ in response to different concentrations of mannitol. Time courses of $[Ca^{2+}]_{cyt}$ (µM) induced by various concentrations of mannitol in barley leaf tips (**A**), root tips (**B**), and Arabidopsis shoots (**C**) and roots (**D**) determined in a luminometer. Dashed lines represent the time point of injection of the different treatments. Values represent means ± SE of three independent replicates.

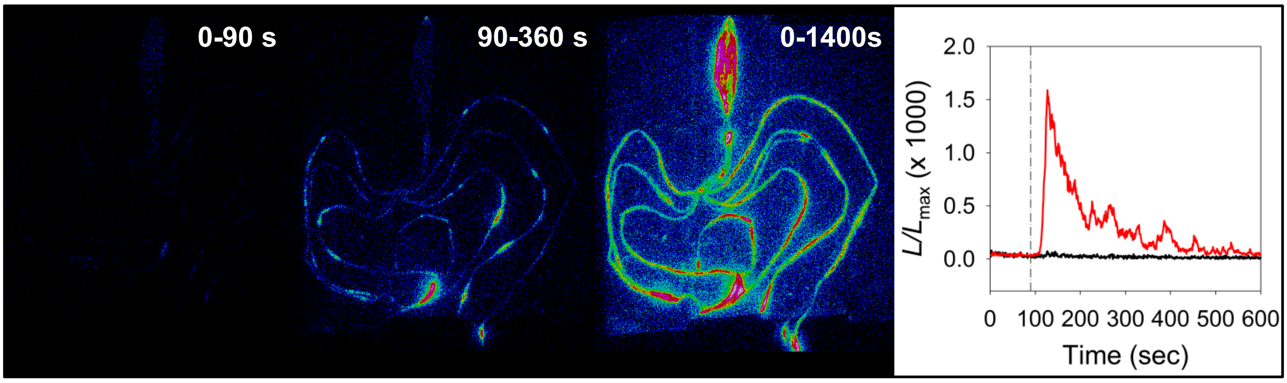

Fig. S5: Root-specificity of the mannitol-induced $[Ca^{2+}]_{cyt}$ elevation in barley. Cumulative images of $[Ca^{2+}]_{cyt}$-dependent photon counts before application of mannitol (0-90 s), in response to 200 mM mannitol (90-360s) or of the entire experiment including a discharge of the whole plant (0-1400s) used to determine L_{max} of each ROI, which represent the entire shoot (black line) or the entire root system (red line). Dashed line represents the time point of mannitol injection.

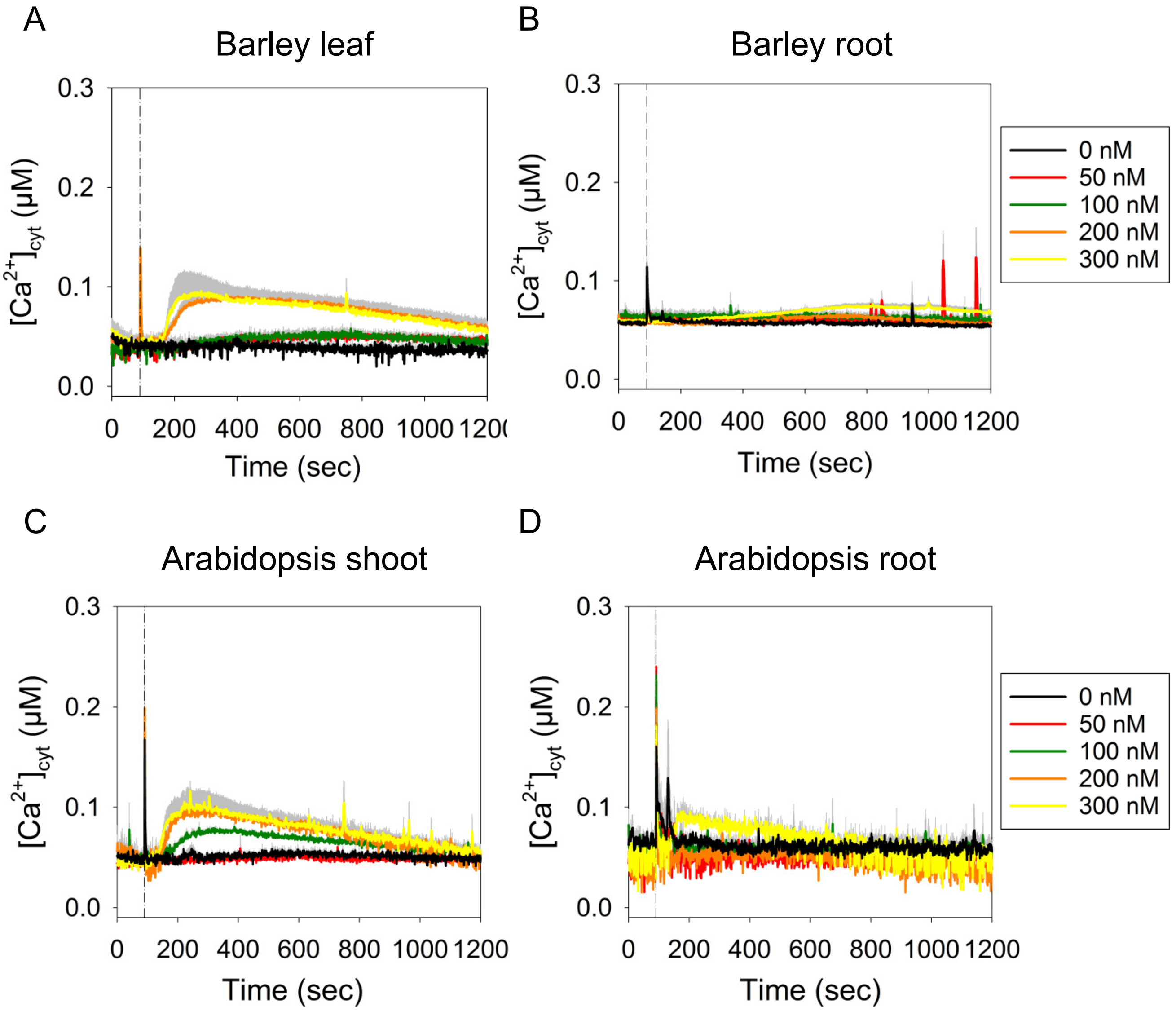

Fig. S6: Kinetics of $[Ca^{2+}]_{cyt}$ in response to different concentrations of flg22. Time courses of $[Ca^{2+}]_{cyt}$ (µM) induced by various concentrations of flg22 in barley leaf tips (**A**), root tips (**B**), and Arabidopsis shoots (**C**) and roots (**D**) determined in a luminometer. Dashed lines represent the time point of injection of the different treatments. Values represent means ± SE of three independent replicates.

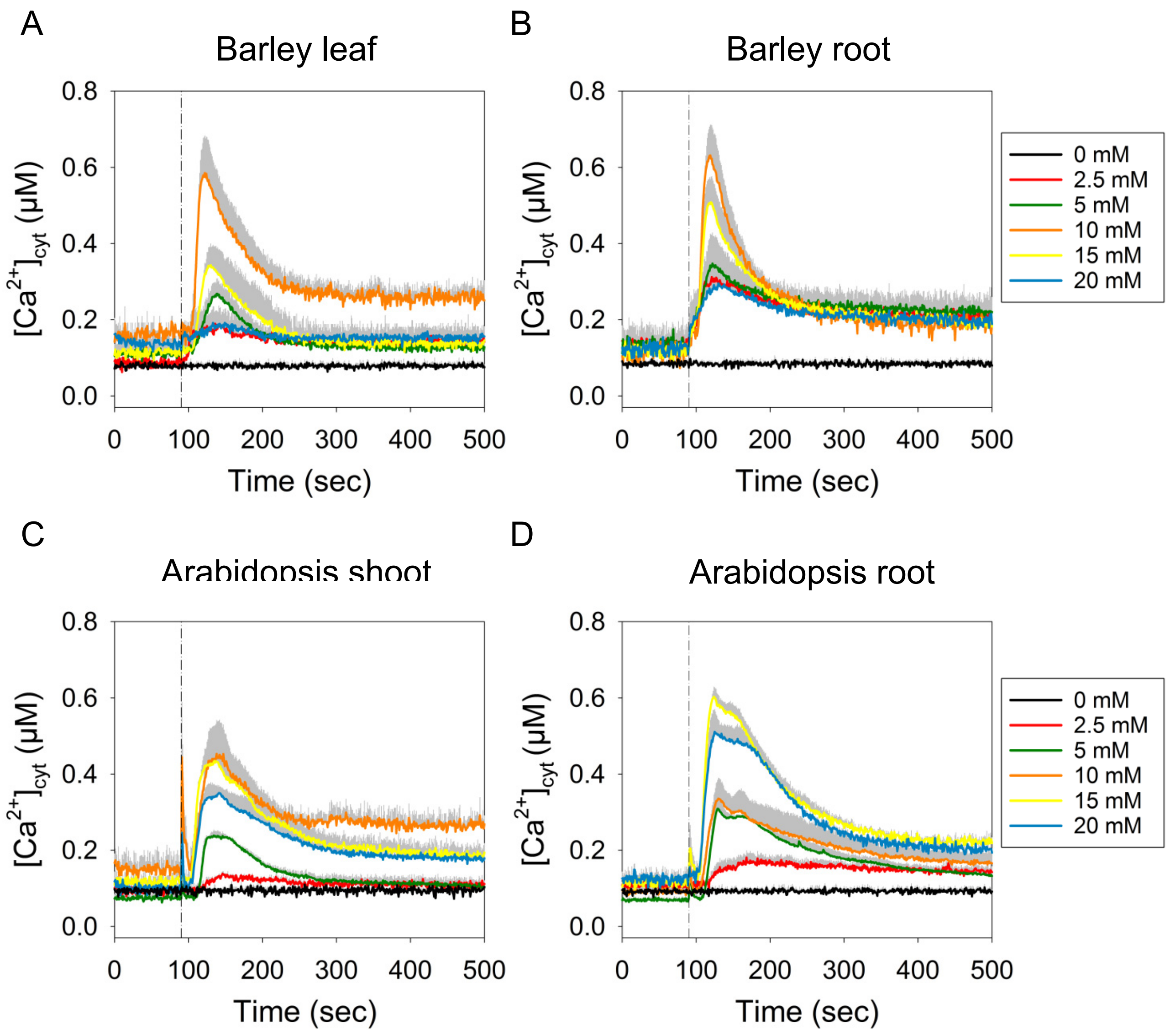

Fig. S7: Kinetics of $[Ca^{2+}]_{cyt}$ in response to different concentrations of H_2O_2. Time courses of $[Ca^{2+}]_{cyt}$ (µM) induced by various concentrations of H_2O_2 in barley leaf tips (**A**), root tips, (**B**) and Arabidopsis shoots (**C**) and roots (**D**) determined in a luminometer. Dashed lines represent the time point of injection of the different treatments. Values represent means ± SE of three independent replicates.

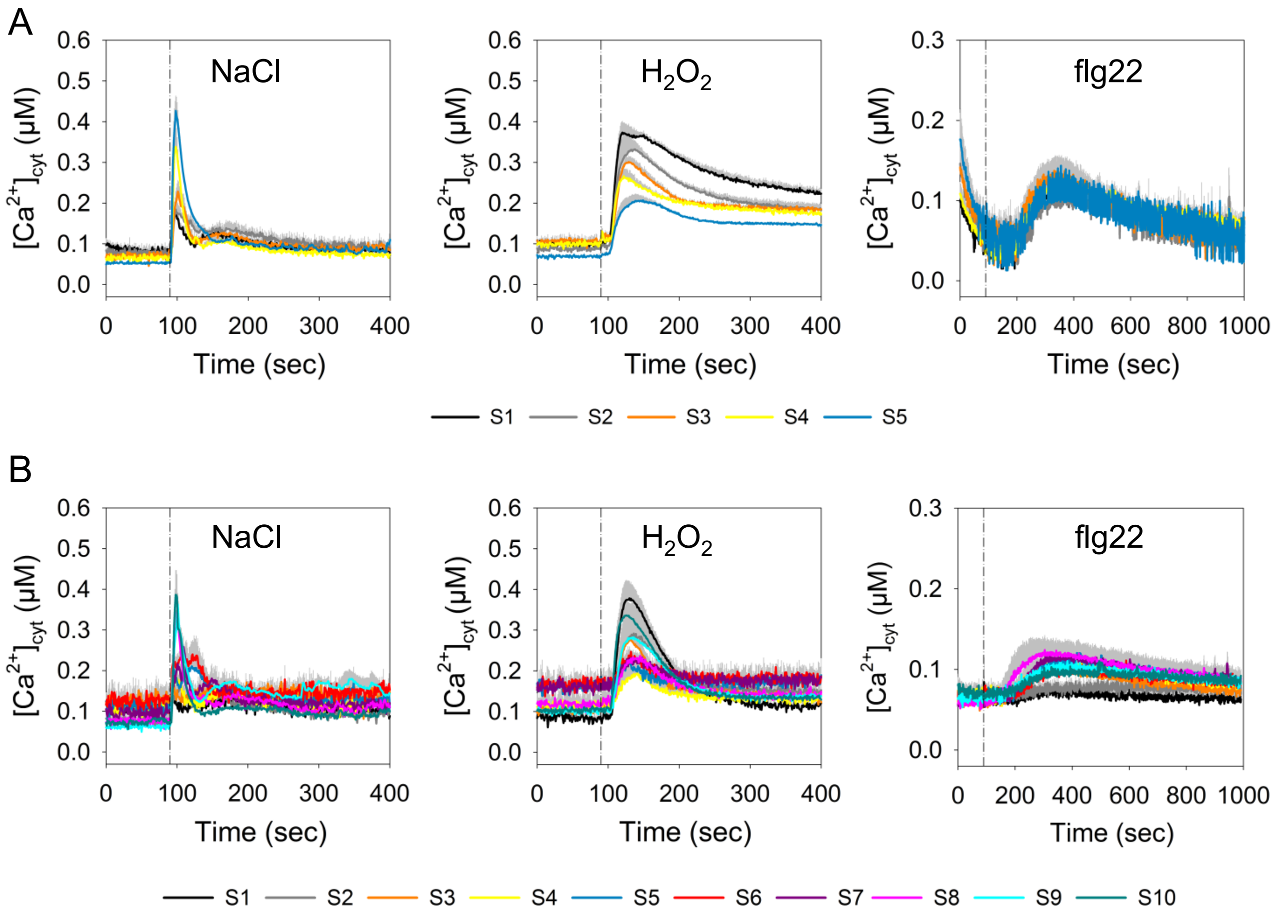

Fig. S8: Kinetics of $[Ca^{2+}]_{cyt}$ in response to NaCl, H_2O_2, and flg22 in sections of five- and seven-day-old barley leaves. Time courses of $[Ca^{2+}]_{cyt}$ (µM) induced by NaCl (250 mM), H_2O_2 (10 mM) and flg22 (200 nM) in sections of five-day-old (**A**) or seven-day-old (**B**) barley leaves determined in a luminometer. Dashed lines represent the time point of injection of the different treatments. Values represent means ± SE of three independent replicates.

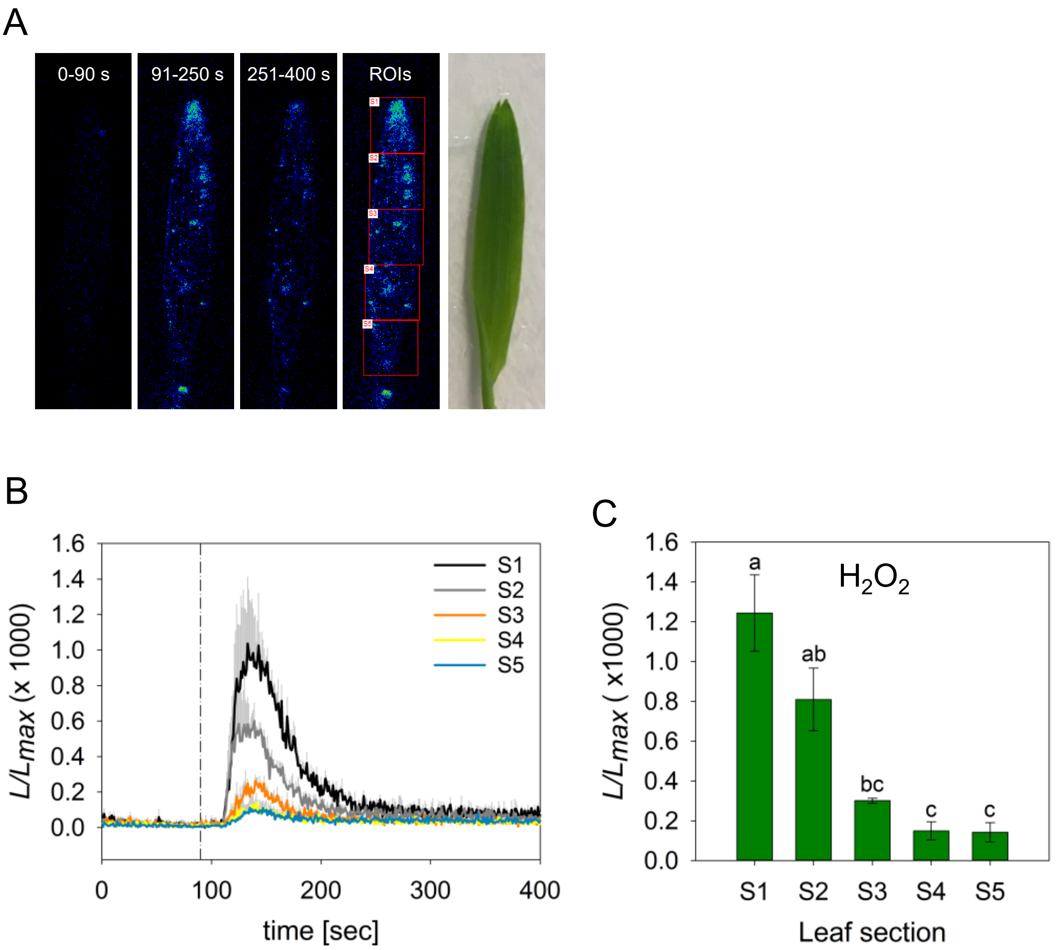

Fig. S9: $[Ca^{2+}]_{cyt}$ -dependent photon counts in response to H_2O_2 in different sections of a five-day-old barley leaf. (A) Cumulative $[Ca^{2+}]_{cyt}$- dependent photon-counting images obtained before (0-90 s), during (91-250 s), and after (251-400 s) treatment with 10 mM H_2O_2 using a photon-counting camera and RGB image of the leaf. ROIs (S1-S5) correspond to the section of the leaves analysed in Fig. 7A. (**B**) Time courses of $[Ca^{2+}]_{cyt}$ changes induced in each ROI of the experiment shown in (**A**). The dashed line represents the time point of H_2O_2 injection. (**C**) Maximal values of photon counts of different sections (ROIs) calculated as *L/Lmax* (see material and methods). Values represent means ± SE of three independent replicates. Letters represent significant differences between the sections according to a One-way ANOVA with Tukey test.
